# Microalgae-blend tilapia feed eliminates fishmeal and fish oil, improves growth, and is cost viable

**DOI:** 10.1038/s41598-020-75289-x

**Published:** 2020-11-12

**Authors:** Pallab K. Sarker, Anne R. Kapuscinski, Brandi McKuin, Devin S. Fitzgerald, Hannah M. Nash, Connor Greenwood

**Affiliations:** 1 Environmental Studies Department, University of California Santa Cruz, Santa Cruz, CA 95064 USA; 2 Health Professions Program, Sciences, Mathematics and Biotechnology, University of California Berkeley Extension, 1995 University Ave., Suite 200, Berkley, CA 94704-7000 USA

**Keywords:** Environmental impact, Ecology, Evolution, Zoology

## Abstract

Aquafeed manufacturers have reduced, but not fully eliminated, fishmeal and fish oil and are seeking cost competitive replacements. We combined two commercially available microalgae, to produce a high-performing fish-free feed for Nile tilapia (*Oreochromis niloticus*)—the world’s second largest group of farmed fish. We substituted protein-rich defatted biomass of *Nannochloropsis oculata* (leftover after oil extraction for nutraceuticals) for fishmeal and whole cells of docosahexaenoic acid (DHA)-rich *Schizochytrium* sp. as substitute for fish oil. We found significantly better (*p* < 0.05) growth, weight gain, specific growth rate, and best (but not significantly different) feed conversion ratio using the fish-free feed compared with the reference diet. Fish-free feed also yielded higher (*p* < 0.05) fillet lipid, DHA, and protein content (but not significantly different). Furthermore, fish-free feed had the highest degree of in-vitro protein hydrolysis and protein digestibility. The median economic conversion ratio of the fish-free feed ($0.95/kg tilapia) was less than the reference diet ($1.03/kg tilapia), though the median feed cost ($0.68/kg feed) was slightly greater than that of the reference feed ($0.64/kg feed) (p < 0.05). Our work is a step toward eliminating reliance on fishmeal and fish oil with evidence of a cost-competitive microalgae-based tilapia feed that improves growth metrics and the nutritional quality of farmed fish.

## Introduction

Aquaculture, the world’s most efficient producer of edible protein, continues to grow faster than any other major food sector in the world, in response to the rapidly increasing global demand for fish and seafood^1,2^. Feed inputs for aquaculture production represent 40–75% of aquaculture production costs and are a key market driver for aquaculture production^1^. The aquafeed market is expected to grow 8–10% per annum and is production of compound feeds is projected to reach 73.15 million tonne (mt) in 2025^2–9^.

Ocean-derived fishmeal (FM) and fish oil (FO) in aquafeeds has raised sustainability concerns as the supply of wild marine forage fish will not meet growing demand and will constrain aquaculture growth^1,2,10,11^. Moreover, competition for FM and FO from pharmaceuticals, nutraceuticals, and feeds for other animals^6,12^ further exacerbates a supply–demand squeeze^2,13^. The use of forage fish (such as herrings, sardines, and anchovies) for FMFO production also affects human food security because approximately 16.9 million of the 29 mt of forage fish that is caught globally for aquaculture feed is directed away from human consumption every year^14^. More than 90 percent of these fish are considered food grade and could be directly consumed by humans, especially food insecure people in developing countries^15^.

Although more prevalent in aquafeeds for high-trophic finfish and crustaceans, FM and FO is also routinely incorporated (inclusion rates of 3–10%) in aquafeeds for low-trophic finfish like tilapia to enhance growth^1,6,16–18^. Tilapia (dominated by *Oreochromis niloticus*)—the world’s second top group of aquaculture organisms—is cultured in such large volumes and is such an integral part of human diets across the world, that even low inclusion rates of FMFO in aquafeeds for this species is a substantial portion of global demand of forage fish (Supplementary Table S1)^19^.

The aquafeed industry reduces reliance on FM and FO by using grain and oilseed crops (e.g., soy, corn, canola), however, terrestrial plant ingredients have low digestibility, anti-nutritional factors, and deficiencies in essential amino acids (lysine, methionine, threonine, and tryptophan)^16,20^. Crop oils also lack long-chain omega-3s (n-3s), eicosapentaenoic acid (EPA) and docosahexaenoic acid (DHA), important for human health^21,22^. Elevated levels of n-6 (e.g. linoleic acid) fatty acids from crop oils^23,24^ changes the long-chain n-3/n-6 ratio in tilapia flesh^25^ that is passed on to human consumers^26–28^, resulting in increased production of pro-inflammatory eicosanoids (via arachidonic acid), which has led nutritionists to doubt the health benefits of farmed tilapia^21,25^.

Alternatives to terrestrial crops have been too costly for broad adoption by aquafeed manufacturers (Sarker et al.^15^). However, nutritional disadvantages and poor fillet quality have prompted researchers to investigate marine microalgae as potential FMFO replacements in fish feeds due to balanced essential amino acids, minerals, vitamins, and long-chain n-3 fatty acids^17,29–38^. The peer-reviewed literature, however, lacks information on how using marine microalgae in fish-free diets affects growth, feed conversion and fillet quality of tilapia. There also are limited published data on the market price of fish-free diets made with alternative ingredients that show potential for economies of scale.

We conducted research to develop a new aquafeed formula by combining the protein-rich (50%) defatted marine microalgal co-products (under-utilized left-over biomass of *Nannochloropsis oculata* after EPA oil extraction for human supplement) with another DHA-rich (30% of total fatty acids) marine microalga (*Schizochytrium *sp.), increasingly available at commercial scale, to fully replace FMFO (fish-free) in tilapia aquafeeds. This study builds on our recent microalgae aquafeeds research. Sarker et al. replaced 33% of FM with under-utilized *N. oculata* defatted biomass in a tilapia diet that achieved final weight, weight gain, percent weight gain, specific growth rate, and protein efficiency ratio values comparable to the reference diet containing FM and FO^17^. Furthermore, it was previously reported that *Schizochytrium* sp. is a highly digestible source of nutrients for tilapia and can fully replace FO in tilapia feed^30,33^.

To examine the commercial viability of using marine microalgae to replace both FM and FO, we conducted a nutritional feeding experiment to compare three microalgal diets to a reference diet containing FM and FO levels found in commercial tilapia feed. Microalgal diets included defatted *N. oculata* to replace 33%, 66% or 100% of FM, and whole cell *Schizochytrium *sp. to replace 100% of FO (33NS, 66NS, 100NS). We measured effects of the four diets on growth metrics, in vitro protein digestibility, feed conversion ratio (FCR), protein efficiency ratio (PER), and fillet deposition of n-3 long-chain polyunsaturated fatty acids (LC PUFAs) and minerals. Furthermore, we conducted a hedonic analysis to estimate the market price of defatted *N. oculata* meal and whole cell *Schizochytrium* sp., feed costs, and the economic feed conversion ratio (ECR).

## Materials and methods

The experimental design and fish use protocol were approved by the Institutional Animal Care and Use Committee (IACUC) of Dartmouth College. Also, we conducted all experiments in accordance with relevant guidelines and regulations. We euthanized the fish by single cranial pithing in the nutritional feeding experiment.

### Diet formulation for nutritional feeding experiment

We incorporated *N. oculata *defatted biomass to replace different percentages of FM and whole cell *Schizochytrium *sp. to replace all FO in three tilapia experimental diets for a nutritional feeding trial. These three diet formulations were based on our previous digestibility data for *N. oculata *defatted biomass and whole cell *Schizochytrium *sp.^17,30,33^, and a prior study showing potential to replace all FO with whole cell *Schizyochytrium* sp.^30^. We compared these three experimental diets to a reference diet (served as control diet) containing FMFO at levels found in commercial tilapia feed. All diets were iso-nitrogenous (37% crude protein) and iso-energetic (12 kJ/g). Microalgae inclusion diets used *N. oculata* defatted biomass to replace 33% (33NS), 66% (66NS), and 100% (100NS) of the FM and whole cell *Schizochytrium *sp. to replace all FO in the test diets (33NS, 66NS, 100NS). Thus *N.* *oculata* comprised 3%, 5% and 8% of the diet by weight, respectively, and *Schizochytrium *sp. made up 3.2% of the diet by weight. We produced the diets in accordance with our previous work^17,30,36^. We obtained dried *Schizochytrium* sp. from ALGAMAC, Aquafauna Bio-marine, Inc., Hawthorne, CA, USA; and menhaden FO from Double Liquid Feed Service, Inc., Danville, IL, USA. Qualitas Health Inc., which markets EPA-rich oil extracted from *N. oculata* as a human supplement^39^ and seeks uses for tons of under-utilized defatted biomass from its large-scale production facilities, donated the *N. oculata* defatted biomass. Supplementary Table S8 reports proximate compositions and amino acid profiles of *N. oculata *defatted biomass and *Schizochytrium *sp*.*; total fatty acid profile by percentage of the defatted biomass and *Schizochytrium *sp ingredients reported in Supplementary Table S9; and macromineral and trace element composition of both ingredients reported in Supplementary Table S10. The formula, proximate analysis, and amino acid profiles of four dietary treatments reported in Table 1. The fatty acid profiles reported in Supplementary Table S11 and the macrominerals and trace elements of the four experimental diets reported in Supplementary Table S7.

**Table 1:** Formulation (g/100 g diet) and essential amino acids (% in the weight of diet) of four experimental diets for juvenile tilapia.

Ingredient (%)	Reference^a^	Diet		
		33NS^b^	66NS^c^	100NS^d^
Fish meal^e^	7	4.69	2.38	0
*N. oculata* defatted biomass	0	3	5.5	8
*Schyzochytrium*	0	6.2	6.2	6.2
Corn gluten meal	30	30	30	30
Soybean meal	30	30	30	30
Wheat flour	20	20	20	20
CaH_2_PO_4_	0.75	0.75	0.75	0.75
Mineral mix^f^	1	1	1	1
Vitamin mix^g^	1	1	1	1
Fish oil	3.2	0	0	0
l-Lysine HCl	0	0.5	0.54	0.6
dl-Methionine	0	0.18	0.2	0.2
Carboxymethyl cellulose	5.07	0.7	0.43	0.27
Choline chloride	2	2	2	2
**Proximate composition (%)**				
Moisture	22.74	19.02	20.06	18.85
Protein	35.01	38.38	37.84	36.78
Fat	5.31	5.56	4.96	5.6
Fiber	1.57	1.78	1.6	1.34
Ash	5.19	5.26	5.14	4.85
Carbohydrates	31.75	31.78	32	33.92
Energy (kJ g^−1^)	11.4	11.9	11.7	12.0
**Amino acids (% in the weight of diet as is)**				
Arginine	2.05	2.25	2.13	2.20
Histidine	1.64	1.70	1.73	1.72
Isoleucine	2.83	2.95	3.11	3.08
Leucine	0.61	0.67	0.65	0.68
Lysine	6.46	6.98	6.82	7.06
Methionine	1.42	1.43	1.34	1.25
Phenylalanine	0.75	0.76	0.71	0.81
Threonine	0.2	0.16	0.11	0.05
Valine	1.05	1.16	1.09	1.28

^a^Reference: no replacement of fish meal (FM) and fish oil (FO).

^b^Replacement of 33% of FM with *N. oculata* and 100% of FO with *Schizochytrium* sp.

^c^Replacement of 66% of FM with *N. oculata* and 100% of FO with *Schizochytrium* sp.

^d^Replacement of 100% of FM with *N. oculata* and 100% of FO with *Schizochytrium* sp.

^e^Omega Protein, Inc. Houston, Texas 77042, as manufacturer specification, the guaranteed gross composition analysis: crude.

protein, 60%; crude fat, 6%; fiber, 2%.

^f^Mineral premix (mg kg^−1^ dry diet unless otherwise stated):ferrous sulphate, 0.13; NaCl, 6.15; copper sulphate, 0.06; manganese , sulphate, 0.18; potassium iodide, 0.02; zinc sulphate, 0.3; carrier (wheat middling or starch).

^g^Vitamin premix (mg kg^−1^ dry diet unless otherwise stated):vitamin A (as acetate), 7500 IU kg^−1^ dry diet; vitamin D3 (as cholecalcipherol), 6000 IU kg^−1^ dry diet; vitamin E (as dl-a-tocopherylacetate), 150 IU kg^−1^ dry diet; vitamin K (as menadione Na-bisulphate), 3; vitamin B12 (as cyanocobalamin), 0.06; ascorbic acid (as ascorbyl polyphosphate), 150; d-biotin, 42; choline (as chloride), 3000; folic acid, 3; niacin (as nicotinic acid), 30; pantothenic acid, 60; pyridoxine, 15; riboflavin, 18; thiamin, 3.

### Experimental design and sampling to evaluate tilapia growth on *N. oculata* defatted biomass and *Schizochytrium *sp. Diets

We conducted the feeding experiment using a completely randomized design of four diets × three replicates tanks in recirculating aquaculture systems (RAS). Four hundred eighty Nile tilapia (mean initial weight 34.5 ± 2.06 g) were put into randomized groups of 40, bulk weighed, and transferred to a tank. Tilapia had been acclimated to the FMFO containing reference diet for 7 days prior to distribution. The initial stocking density remained within levels recommended to avoid physiological stress on tilapia (< 0.25 lbs/gal in 80 gallon RAS tanks). We carefully monitored water quality daily to maintain favorable conditions for tilapia across all RAS tanks and kept the water temperature at 28.7 ± 0.25 °C, pH at 7.1 ± 0.1, dissolved oxygen at 6.1 ± 0.15 mg/L, total ammonia nitrogen at 0.26 ± 0.1 mg/L, and nitrite nitrogen at 0.3 ± 0.01 mg/L^17,30^.

We administered feed at a rate of 8% of body weight until day 60, 6% until day 121, and 4% until day 183, with feedings performed twice per day at 09:00 and 15:30 h. We measured fish biomass monthly by randomly selecting 10 fish as a weight sample to adjust feeding rates for growth and we bulk weighed all fish every other month for sampling events (day 0, 60, 121, and 185). We withheld feed for 24 h prior to the weighing procedure to reduce handling stress on fish.

### Biological sampling and tissue collection

We randomly selected and weighed 10 individual fish from the total starting stock at the beginning of the experiment, then euthanized (by single cranial pithing^17^, and stored fish tissues at – 20 °C for future biochemical analysis. At day 121 of the experiment, we euthanized 6 fish per tank, and 6 additional fish at day 185, the terminus of the trial. Half of the fish sampled on day 121 and day 185 were filleted, and half were kept whole and then stored at − 20 °C for further processing^17,30^. All samples from the initial sampling, day 121, and day 185 were freeze dried at − 20 °C, then fully homogenized. Both whole body and fillet samples were sent to New Jersey Feed Laboratory, Inc (Ewing, NJ, USA) for full proximate, energy, amino acid, and fatty acid profiles.

### Analytical procedure and calculation

We quantified final weight, weight gain, weight gain percentage, FCR, SGR, PER, and survival rate for each of the dietary treatments. Each of these parameters were calculated as follows: weight gain = (final weight − initial weight/initial weight) × 100; FCR, FCR = feed intake/weight gain; protein efficiency ratio; SGR (%/day) = 100 × ln final wet weight (g) − ln initial wet weight (g))/Time (days), PER = weight gain (g)/protein fed (g); and survival rate (%) = (final number of fish/initial number of fish) × 100^17,34,40^.

The trace mineral content of each of the experimental diets, sampled fish fillets, and whole bodies was analyzed by the Department of Earth Science at Dartmouth College^17^. Each 100 mg sample was acid digested in 0.5 mL 9:1 HNO_3_/HCl in open vessel digestion with heating at 105 °C for 1 h. Samples were diluted to 10 mL in DI water prior to analysis. All measurements were recorded gravimetrically. Digested samples were run by ICP-MS analysis using an Agilent 7700 × with collision (He) and reaction (H_2_) gases. The methodology and quality control followed EPA method 6020a.

### Degree of protein hydrolysis and in-vitro protein digestibility

We performed an in-vitro digestibility assessment according to the method prescribed in Yasumaru and Lemos to measure the degree of protein hydrolysis of our experimental diets in the presence of tilapia stomach crude enzyme extract and intestine crude enzyme extract^41^. A 50 g sample from each of the four diets was ground via mortar and pestle until all materials could fit through a 0.5 mm food sieve. We allotted 80 mg by protein basis of each diet with 25 mL DI water in a 50 mL reaction vessel immersed in a water bath held at 25 °C. The reaction mixture, containing diet and DI water, was adjusted to pH 2.0 with 0.1 M HCl using a Hannah instrument HI-901C1 potentiometric auto titrator, set to dose 0.3 mL HCl every 2 min for 30 min until pH equilibrium was reached. After equilibrium, we introduced 200 µL stomach crude enzyme extract prepared according to Yasumaru and Lemos with storage solution modifications sourced from Chaijaroen and Thongruang^41,42^. After crude enzyme extract introduction, we made minor pH changes adding 0.1 M HCl or 0.01 M NaOH by hand when necessary. Once we introduced the crude enzyme extract, we initiated a predetermined program on the auto titrator to dose 0.025–0.075 mL in proportion to the change in pH measured. This program dosed accordingly every 3-min interval to keep the pH at 2.0 for 1 h. The program was paused, when necessary, to prevent over adjusting the solution during the titration. After the 1-h stomach digestion period, we recorded the total volume dosed. We then adjusted the reaction mixture pH to 8.0, using 0.1 M NaOH, and allowed the auto titrator to dose 0.025 mL 0.1 M NaOH for approximately 1 h to allow the mixture to reach equilibrium. Once pH equilibrium was reached, we introduced 250  µL intestinal crude enzyme extract, prepared in the same way as the stomach crude enzyme extract. Minor adjustments to pH were made by hand using 0.01 M NaOH or 0.1 M HCl. Then we initiated the auto titrator method to dose 0.01–0.025 mL 0.1 M NaOH proportional to the measured change in pH, in order to hold the pH at 8.0 for 1 h, and recorded the total volume dosed. All diets were run in triplicate^41,43^. We quantified the degree of protein hydrolysis in the stomach using the following equation:

1 $$DH = \left[ {\frac{V \times N}{E}} \right] \times \left( \frac{1}{P} \right) \times F_{pH} \times 100\% ,$$

where DH is the degree of hydrolysis, V is the volume of the acid consumed (mL), N is the normality of the acid (H^+^ available for release × Molarity), E is the mass of the substrate protein (g), P is the number of peptide bonds cleaved (mol g protein^−1^) and when amino acid composition is unknown, (8.0), and F_pH_ is the correction factor for pH 2.0 at 25 °C (1.08).

We quantified the degree of protein hydrolysis in the intestine using the following equation:

2 $$DH = B \times Nb \times \left( \frac{1}{a} \right) \times \left( \frac{1}{MP} \right) \times \left( {\frac{1}{{H_{tot} }}} \right) \times 100\% ,$$

where B is the volume of alkali consumed (mL), Nb is the normality of the alkali (alkali groups × Molarity), a is the average degree of dissociation of the a-NH_2_ groups (1/a = 1.50 for pH 8.0 at 25 °C), MP is the mass of substrate protein (g), and H_tot_ is the total number of peptide bonds in the protein substrate [7.6–9.2 meqv g protein^−1^] according to the source of protein^44^.

After calculating the degree of protein hydrolysis, we determined the in vitro protein digestibility using a prediction equation model as reported by Yasumaru and Lemos and Tibbets^41,43^. The degree of protein hydrolysis was used as input in the following equation to determine in vitro protein digestibility, IPD = (3.5093DH + 70.248).

### Economic analysis of fish-free feed formulated with microalgae blends

We obtained commodity and market prices for the formulated feed ingredients from a variety of sources (Supplementary Tables S5 and S12). We conducted non-parametric bootstraps in RSTUDIO (v.1.2.5033) based on 10,000 replicates using the adjusted bootstrap percentile method to estimate the median and 95% confidence intervals.

We conducted a hedonic analysis in RSTUDIO to estimate the price of defatted *N. oculata* meal and whole cell *Schizochytrium* sp. The general methodology of hedonic analysis is described in Maisashvili et al.^45^. We used mixed-effects linear models using maximum likelihood methods^46,47^.

Following Maisashvili et al., we selected crude protein, ether extract, methionine, and lysine as the key input variables in our defatted *N. oculata* meal model^45^. We used the following regression formula:

3 $${\varvec{y}}_{t} = \beta_{0} + b_{{0,CP_{t} }} + b_{{0,EE_{t} }} + \beta_{1} \cdot {\varvec{CP}}^{2} + \beta_{2} \cdot {\varvec{Met}}^{2} + \beta_{3} \cdot {\varvec{Lys}}^{2} + b_{{1_{t} }} \cdot {\varvec{CP}} + \left( {\beta_{4} + b_{{2_{t} }} } \right) \cdot {\varvec{EE}} + \varepsilon ,$$

where **y**_t_ is the vector of feed ingredient prices observed at time t, **CP** is a vector of independent variables reflecting the crude protein content of the corresponding feed ingredients, **Met** is a vector of independent variables reflecting the methionine content of the corresponding feed ingredients, **Lys** is a vector of independent variables reflecting the lysine content of the corresponding feed ingredients, **EE** is a vector of independent variables reflecting the ether extract content of the corresponding feed ingredients, β_0_ is the fixed-effect intercept, β_1_ is the fixed-effect coefficient of **CP**^2^, β_2_ is the fixed-effect coefficient of **Met**^2^, β_3_ is the fixed-effect coefficient of **Lys**^2^, β_4_ is the fixed-effect coefficient of **EE**, b_0,CP_ is the random-effect intercept of **CP** at time t, b_0,EE_ is the random-effect intercept of **EE** at time t, b_1_ is the random-effect coefficient of **CP** at time t, b_2_ is the random-effect coefficient of **EE** at time t, ε is the residual error, and t is the time period (2010–2019).

We selected the top fatty acids present in both the commodity oils (vegetable and fish) and in *Schizochytrium* sp. that did not require an extrapolation. Thus, we used the following regression formula:

4 $${\varvec{y}}_{t} = \beta_{0} + b_{{0,14:0_{t} }} + b_{{0,16:0_{t} }} + \beta_{1} \cdot {\user2{20:5}}{\mathbf{n}}{ - }{\user2{3}}^{2} + \beta_{2} \cdot {\user2{14:0}}^{2} + \beta_{3} \cdot {\user2{16:1}}{\varvec{n}}{ - }{\user2{7}}^{2} + \left( {\beta_{4} + b_{{1_{t} }} } \right) \cdot {\user2{14:0}} + \left( {\beta_{5} + b_{{2_{t} }} } \right) \cdot {\user2{16:0}} + \varepsilon ,$$

where **y**_t_ is the vector of oil ingredient prices observed at time t, **20:5n-3** is a vector of independent variables reflecting the EPA content of the corresponding oil ingredients,** 14:0** is a vector of independent variables reflecting the myristic acid content of the corresponding oil ingredients, **16:1n-7** is a vector of independent variables reflecting the palmitoleic acid content of the corresponding oil ingredients, **16:0** is a vector of independent variables reflecting the palmitic acid content of the corresponding oil ingredients, β_0_ is the fixed-effect intercept, β_1_ is the fixed-effect coefficient of **20:5n-3**^2^, β_2_ is the fixed-effect coefficient of **14:0**^2^, β_3_ is the fixed-effect coefficient of **16:1n-7**^2^, β_4_ is the fixed-effect coefficient of **14:0**, β_5_ is the fixed-effect coefficient of **16:0,** b_0,14:0_ is the random-effect intercept of **14:0** at time t, b_0,16:0_ is the random-effect intercept of **16:0** at time t, b_1_ is the random-effect coefficient of **14:0** at time t, b_2_ is the random-effect coefficient of **16:0** at time t, ε is the residual error, and t is the time period (2010–2019).

As inputs to Eqs. (3) and (4), we used the mean annual prices for 12 meal ingredients and 7 oil ingredients from January 2010 to December 2019 (see Supplementary Table S12 for details about the commodities and data sources). Although some studies have used shorter time horizons for their hedonic models (e.g. 2 years)^48^, we followed other studies that used longer time horizons (e.g. 10 years) in their hedonic models^49^ and economic analysis of agricultural commodities to capture variability^50^. We incorporated a freight component to calculate the costs to bring these commodities to the Port of Shanghai, China. To account for the multi-modal components of the freight costs of U.S. commodities, we applied modal transport shares (e.g. rail, truck, barge) of grain commodities (e.g. corn, wheat, soybeans, sorghum, and barley) to the distances between the grain production sites and U.S. ports (see Supplementary Table S13 and Supplementary Methods for further details). We used a shipping route distance calculator to estimate the international shipping distances (Supplementary Table S14). We obtained the nutritional composition of the feed commodities from Archer Daniel Midlands and Feedinamics (Supplementary Table S15). We obtained the fatty acid profiles of the oils used in the feed from the literature (Supplementary Table S16). For the terrestrial-plant-based oils, we used the fatty acid values reported in Dubois et al.^51^. For FO, we used the fatty acid values reported in Sarker et al.^30^. We scaled the vectors of independent variables (Supplementary Tables S15 and S16) with the parameters provided in Supplementary Tables S17 and S18, for defatted *N. oculata* and whole cell *Schizochytrium* sp., respectively. We assessed the goodness of fit using graphical methods and diagnostic tests (see Supplementary Methods, Supplementary Tables S19 and S20, and Supplementary Figs. S2–S7 for further details).

We estimated the price of defatted *N. oculata* meal with Eq. (3), the scaled parameters (Supplementary Table S21), the fixed-effect coefficients (Supplementary Table S22), and the random-effect coefficients (Supplementary Table S23). We estimated the price of whole cell *Schizochytrium* sp. with Eq. (4), the scaled parameters (Supplementary Table S24), the fixed-effect coefficients (Supplementary Table S25), and the random-effect coefficients (Supplementary Table S26). To convert the estimated price of *Schizochytrium* sp. oil to whole cell *Schizochytrium* sp., we multiplied the price by the fraction of lipids in *Schizochytrium* (0.54).

We calculated the costs of all ingredients of formulated reference feed and experimental feeds (which combined *N. oculata* defatted biomass with *Schizochytrium *sp.) to determine the diet costs in USD per kg (Supplementary Table S27). The price of each diet was determined by multiplying the respective contributions of each feed ingredient by their respective costs per kg and summing the values obtained for all of the ingredients in each of the formulated diets. Finally, we estimated the production cost of tilapia ($/kg fish) via ECR to compare among the four experimental tilapia feeds (which combined defatted biomass with *Schizochytrium* sp.). We estimated fish production cost as ECR using the equation of Piedecausa^52^:

5 $$ECR \left( {\frac{\$ }{{{\text{kg}}\,fish}}} \right) = FCR \left( {\frac{{{\text{kg}}\, diet\, fed}}{{{\text{kg}} \,weight \,gain}}} \right) \times price \,of\, diet \left( {\frac{USD\$ }{{{\text{kg}} \,diet}}} \right),$$

where ECR is the economic conversion ratio, and FCR is the feed conversion ratio.

### Statistical analysis

Statistical analysis (ANOVA) was performed according to Sarker et al.^17^ to determine the significant differences in proximate and amino acid content, fatty acid profile, final weight, weight gain, weight gain percentage, in vitro protein digestibility, FCR, SGR, PER, survival rate, and ECR for each of the treatments. When significant differences were found, we compared the treatment means using Tukey’s test of multiple comparisons (posthoc), with a 95% confidence interval. The IBM Statistical Package for the Social Sciences (SPSS) program for Windows (v. 21.0, Armonk, NY, USA) was used for all statistical methods.

### Data and code availability

The datasets and RSTUDIO files used in the economic analysis including the hedonic regression analyses (used to estimate the price of defatted *N. oculata* meal and whole cell *Schizochytrium*), bootstrap confidence intervals of feed ingredient prices, and the ECR for Fig. 2 are available at the following link: 10.6071/M3VD5V.

## Results

### Growth, nutrient utilization and proximate composition of tilapia carcass

Fish fed the fish-free diet for 184 days displayed significantly better (*p* < 0.05) final weight, weight gain, percent weight gain and specific growth rate than fish fed the reference diet, which contained FM and FO levels typically found in commercial tilapia diets (Table 2). Growth rates were linear throughout the experiment and weights measured for the fish-free diet diverged from those for the reference diet by day 128 (Supplementary Fig. S1). Tilapia fed fish-free feed showed an improved food conversion ratio and protein use efficiency ratio though differences among diets and were not statistically significant. We detected no difference in survival rate among all diets and all fish appeared healthy (no visual signs of illness or deformities) at the end of the experiment. The whole-body proximate composition (Supplementary Table S2) did not significantly differ across the dietary treatments; lipid contents ranged from 2 to 5% and protein contents ranged from 13 to 17% across the four treatments.

**Table 2:** Results from feeding tilapia iso-nitrogenous, iso-caloric, iso-energetic diets that replaced different percentages of fish meal with *N. oculata* defatted biomass and of fish oil with *Schizochytrium* sp*.* whole cells.

	Diet^a^				ANOVA	
	Reference^b^	33NS^c^	66NS^d^	100NS^e^	*F* value	*P* value
Initial Wt. (g)	33.3 ± 1.7	35.5 ± 2.2	34.9 ± 2.1	34.4 ± 2.2	0.2	0.88
Final Wt. (g)	139.9 ± 4.5^f^	196.1 ± 23.6^f,g^	168.9 ± 19.9^f,g^	207.3 ± 9.8^g^	4	0.05
Wt. gain (g)^h^	106.6 ± 13.1^f^	160.6 ± 21.4^f,g^	135.8 ± 4.6^f,g^	172.9 ± 8.4^g^	4.7	0.03
Wt. gain (%)^i^	318.8 ± 28.0^f^	447.8 ± 34.6^f,g^	392.6 ± 27.7^f,g^	504.3 ± 27.3^g^	7.2	0.01
FCR^j^	1.61 ± 0.1	1.57 ± 0.1	1.60 ± 0.1	1.40 ± 0.1	3	0.09
SGR^k^	0.62 ± 0.05^f^	0.81 ± 0.04^f,g^	0.74 ± 0.04^f,g^	0.87 ± 0.03^g^	6.5	0.01
PER^l^	1.23 ± 0.1	1.1 ± 0.0	1.1 ± 0.1	1.3 ± 0.02	2.9	0.1
Survival (%)^m^	93.3 ± 1.7	93.33 ± 0.8	97.5 ± 1.4	90.8 ± 5.5	0.8	0.49

^a^Values are means ± standard errors of three replicate groups (n = 3).

^b^Reference: no replacement of fish meal (FM) and fish oil (FO).

^c^Replacement of 33% of FM with *N. oculata* and 100% of FO with *Schizochytrium* sp.

^d^Replacement of 66% of FM with *N. oculata* and 100% of FO with *Schizochytrium* sp.

^e^Replacement of 100% of FM with *N. oculata* and 100% of FO with *Schizochytrium* sp.

^f, g^Mean values not sharing a superscript letter in the same row differ significantly (P < 0.05) from Tukey’s HSD test.

^h^Weight (Wt.) gain (g) = final Wt. − initial Wt.

^i^Wt. gain (%) = (final Wt. − initial Wt.)/initial Wt. × 100.

^j^Feed conversion ratio (FCR) = feed intake (g)/Wt. gain (g).

^k^Specific growth rate (SGR) (%/day) = 100% × (ln final wet Wt. (g) − ln initial wet Wt. (g))/Time (days).

^l^Protein efficiency ratio (PER) = Wt. gain (g)/protein fed (g).

^m^Survival (%) = (Final number of fish/Initial number of fish) × 100%.

### Fillet proximate and amino acid composition

We detected the highest crude protein, lipid, and ash content in the fillet tissue of tilapia fed the fish-free feed (100NS), with the only significant difference (*p* < 0.05) being crude lipid (Supplementary Table S3). Crude protein contents ranged from 18–24% among the four dietary treatments. Nile tilapia fillets from the fish-free feed treatment had significantly higher lipid content (1.8%) compared to fillets from the reference (0.8%), 33NS, (0.9%), and 66NS (0.9%) feeds. The fillet amino acid composition, except for methionine and histidine, did not differ across the diets (Supplementary Table S4). We detected significantly lower (*p* < 0.05) methionine and histidine content in the 33NS diet compared to other diets. Methionine and histidine content in the 66NS diet was the highest when compared to the fish-free and reference diets, but was not significantly different.

### Fillet macro minerals and trace elements composition

We did not find any significant differences in macromineral composition in fillets across all diets (Table 3). Fillet trace element composition also did not significantly differ across the dietary treatments, except for selenium, which differed significantly (*p* < 0.05) between the reference and 33NS diets but not among the reference, fish-free and 66 NS diets. We detected the lowest level of arsenic in fish fillet of fish-free feed. Other trace elements—boron, mercury, lead and molybdenum—were at non-detectable levels in all fish fillets.

**Table 3:** Macro minerals and trace elements content (wet weight basis) of fillet from Nile tilapia after 184 days on the experimental diets.

Macro minerals (%)	Reference^b^	Fillet^a^			ANOVA	
		33NS^c^	66NS^d^	100NS^e^	*F* Value	*P* Value
Phosphorus	11.92 ± 1.89	10.85 ± 0.78	12.98 ± 0.24	9.53 ± 0.84	1.75	0.23
Calcium	7.37 ± 2.45	6.43 ± 1.01	8.53 ± 0.73	3.08 ± 0.84	2.65	0.12
Magnesium	1.28 ± 0.14	1.19 ± 0.06	1.37 ± 0.06	1.28 ± 0.12	0.58	0.64
Potassium	18.19 ± 2.33	16.78 ± 0.47	18.83 ± 0.99	17.65 ± 1.55	0.33	0.8
Sulfur	10.87 ± 0.84	10.21 ± 0.3	11.65 ± 0.62	10.51 ± 0.26	1.25	0.35
**Trace elements (mg kg**^**−1**^**)**						
Copper	1.74 ± 0.11	1.47 ± 0.17	1.8 ± 0.21	1.65 ± 0.2		
Iron	17.43 ± 1.58	14.28 ± 1.07	19.51 ± 1.29	16.83 ± 1.15	2.8	0.1
Manganese	0.91 ± 0.13	0.75 ± 0.07	0.87 ± 0.08	0.67 ± 0.05	1.67	0.24
Selenium	0.95 ± 0.08^f^	0.63 ± 0.05^g^	0.84 ± 0.04^f,g^	0.75 ± 0.04^f,g^	6.73	0.01
Zinc	42.28 ± 10.92	32.91 ± 0.59	38.69 ± 1.3	33.46 ± 2.36	0.62	0.61
Arsenic	0.21 ± 0.04	0.14 ± 0.03	0.27 ± 0.03	0.14 ± 0.02		
Boron	ND^h^	ND	ND	ND		
Aluminum	ND	ND	ND	0.01		
Mercury	ND	ND	ND	ND		
Lead	ND	ND	ND	ND		
Molybdenum	0.07 ± 0	0.06 ± 0.01	0.08 ± 0.01	0.08 ± 0.01		

^a^Values are means ± standard errors of three replicate groups (n = 3); each replicate involving pooled whole tissues of 5 fish.

^b^Reference: no replacement of fish meal (FM) and fish oil (FO).

^c^Replacement of 33% of FM with *N. oculata* and 100% of FO with *Schizochytrium* sp.

^d^Replacement of 66% of FM with *N. oculata* and 100% of FO with *Schizochytrium* sp.

^e^Replacement of 100% of FM with *N. oculata* and 100% of FO with *Schizochytrium* sp.

^f, g^Mean values not sharing a superscript letter in the same row differ significantly (P < 0.05) from Tukey’s HSD test.

^h^Not detectable (ND) (< 0.000 µg/g).

### Fillet fatty acid (% of total fatty acids) content

The fillet of tilapia fed the experimental diets was similar to the dietary fatty acid content of the corresponding feed. Across diets, the concentrations of total n-3 PUFA, n-6 PUFA, n-3 LC PUFA, and n-6 LC PUFA, were not significantly different (Table 4). We also found that the total saturated fatty acid (SFA), most of the SFA fractions, total mono-unsaturated fatty acids (MUFA), and most MUFA fractions did not differ across diets. Fish fed the reference diet displayed the highest (*p* < 0.05) concentrations of 16:1n-7 which corresponds to the 16:1n-7 content in experimental diets. In the fillet of fish fed the reference and fish-free feed, we detected similar MUFA fractions of 16:1n-9, 18:1n-7, and 20:1n-9. Total PUFAs were significantly higher (*p* < 0.05) in tilapia fillet fed microalgae inclusion diets (33NS, 66NS, and 100NS) compared to the reference diet. Many of the individual PUFAs did not vary greatly among dietary treatments. However, n-6 fatty acids, 18:3n-6, 20:3n-6, 22:4n-6, and 22:5n-6 showed significant differences (*p* < 0.05) between the diets. Among n-3 PUFAs, we detected significantly higher (*p* < 0.05) 22:6n-3 DHA in tilapia fed microalgae inclusion diets compared to reference diet. The highest EPA content in the reference diet reflected the higher EPA supplied by this diet. The reference diets had the highest concentrations of 20:3n-6, and 22:4n-6 compare to the three other treatments. In contrast, tilapia fed the reference diet had significantly (*p* < 0.05) decreased concentrations of 22:5n-6 compared to fish fed microalgae inclusion diets. The n-3/n-6 PUFA ratios did not differ significantly between all four dietary treatments. The n-3/n-6 LC PUFA ratio was highest in the fish-free and reference diets.

**Table 4:** Fatty acid content of fillets from Nile tilapia after 156 days on the experimental diets.

Fillet (% TFA)^a^	Reference^b^	33NS^c^	66NS^d^	100NS^e^	*F* value	*P* value
14:00	3.75 ± 0.09	2.8 ± 0.06	3.57 ± 0.53	4.3 ± 0.47	2.96	0.09
15:00	0.39 ± 0.02	0.26 ± 0.01	0.36 ± 0.07	0.38 ± 0.05	1.55	0.27
16:00	19.71 ± 0.38^f,g^	16.38 ± 0.12^g^	22.32 ± 1.84^f,g^	23.8 ± 2.03^f^	5.49	0.02
17:00	0.45 ± 0.04^f^	0.26 ± 0.02^g^	0.36 ± 0.03^f,g^	0.31 ± 0.03^g^	6.74	0.01
18:00	7.2 ± 0.06^f^	5.26 ± 0.08^g^	7.43 ± 0.42^f^	6 ± 0.35^f,g^	13.38	0
20:00	0.36 ± 0.03	0.26 ± 0.01	0.31 ± 0.04	0.32 ± 0.02	2.73	0.11
22:00	0.14 ± 0.07	0.08 ± 0.04	0.14 ± 0.02	0.12 ± 0	0.49	0.69
24:00	0.2 ± 0.05	0.15 ± 0.02	0.25 ± 0.06	0.09 ± 0.05	1.18	0.57
Total SFA^i^	32.2 ± 2.41	25.45 ± 2	34.74 ± 2.72	35.32 ± 2.88	2.01	0.19
16:1n-9	0.53 ± 0.05^f^	0.34 ± 0.01^g^	0.39 ± 0.04^f^	0.42 ± 0.03^f^	5.3	0.02
16:1n-7	3.93 ± 0.05^f^	1.64 ± 0.03^h^	2.2 ± 0.44^f,g,h^	3.4 ± 0.5^f,g^	9.99	0
18:1n-9	19.03 ± 0.39	12.91 ± 0.08	16.3 ± 2.11	18.06 ± 1.98	3.41	0.07
18:1n-7	3.15 ± 0.13^f^	1.82 ± 0.04^g^	2.51 ± 0.18^f^	2.65 ± 0.21^f^	12.34	0
20:1n-9	1.23 ± 0.07^f^	0.67 ± 0.02^g^	0.83 ± 0.11^f,g^	0.92 ± 0.08^f^	9.88	0
20:1n-7	0.04 ± 0.04	0 ± 0	0 ± 0	0 ± 0	1	0.44
22:1n-11	0.19 ± 0.11	0.02 ± 0.02	0.21 ± 0.04	0.14 ± 0.08		
22:1n-9	0.05 ± 0.05	0.04 ± 0.04	0.23 ± 0.14	0.14 ± 0.14	0.74	0.55
24:1n-9	0 ± 0	0 ± 0	0 ± 0	0 ± 0	2.29	0.155
Total MUFA^j^	28.15 ± 2.29	17.44 ± 1.56	22.67 ± 1.96	25.73 ± 2.18	3.6	0.06
18:2n-6	13.13 ± 0.22	9.66 ± 0.14	10.95 ± 0.51	11.61 ± 0.51		
18:3n-6	0.52 ± 0.09^f^	0.16 ± 0.02^g^	0.18 ± 0.03^f,g^	0.21 ± 0.06^f^	9.27	0
20:2n-6	0.72 ± 0.07	0.59 ± 0.03	0.68 ± 0.05	0.62 ± 0.11	0.68	0.58
20:3n-6	0.96 ± 0.01^f^	0.43 ± 0.02^g^	0.51 ± 0.05^g^	0.5 ± 0.09^g^	22.31	0
20:4n-6 ARA^k^	2.92 ± 0.15	1.8 ± 0.03	2.53 ± 0.65	1.68 ± 0.3	2.62	0.12
22:4n-6	0.9 ± 0.04^f^	0.38 ± 0.02^g^	0.43 ± 0.11^g^	0.42 ± 0.13^g^	7.8	0
22:5n-6	1.22 ± 0.02^g^	4.98 ± 0.13^f^	6.15 ± 1.64^f^	4.79 ± 1.19^f^	4.4	0.04
Total n-6 PUFA^l^	20.37 ± 1.73	18 ± 1.34	21.43 ± 1.54	19.83 ± 1.58	0.15	0.92
18:3n-3 ALA^m^	0.73 ± 0.07	0.52 ± 0.02	0.5 ± 0.05	0.58 ± 0.07	3.54	0
18:4n-3	0.22 ± 0.02	0 ± 0	0.0 ± 0.0	0 ± 0		
20:3n-3	0.16 ± 0	0.1 ± 0.01	0.07 ± 0.04	0.18 ± 0.04	3.12	0.08
20:4n-3	0.29 ± 0.02^f^	0.12 ± 0.01^f,g^	0.08 ± 0.04^g^	0.12 ± 0.06^f,g^	5.38	0.02
20:5n-3 EPA^n^	1.47 ± 0.27^f^	0.33 ± 0.02^†^	0.46 ± 0.1^g^	0.35 ± 0.08^g^	13.63	0.02
22:5n-3	2.92 ± 0.11	0.77 ± 0.02	0.94 ± 0.2	1.05 ± 0.24		
22:6n-3 DHA^o^	7.02 ± 0.47^g^	10.17 ± 0.15^f^	11.99 ± 2.7^f^	10.59 ± 2.7^f^	36.45	0
Total n-3 PUFA^p^	12.81 ± 0.94	12.01 ± 1.41	14.07 ± 1.67	12.87 ± 1.46	8	0
Total PUFA	33.18 ± 2.67^g^	30.01 ± 2.75^f^	35.5 ± 3.21^f^	32.7 ± 3.04^f^	0.86	0.5
Total n-6 LCPUFA^q^	6.72 ± 0.29	8.18 ± 0.23	10.3 ± 2.5	8.01 ± 1.82	0.94	0.46
Total n-3 LCPUFA^r^	11.86 ± 0.87	11.49 ± 0.21	13.54 ± 3.08	12.29 ± 3.12	0.16	0.91
n-3/n-6 PUFA ratio^s^	0.63 ± 0.54	0.67 ± 1.05	0.66 ± 1.08	0.65 ± 0.92	0.07	0.97
n-3/n-6 LCPUFA ratio	1.76 ± 3^f^	1.4 ± 0.91^g^	1.31 ± 1.23^g^	1.53 ± 1.71^f^	8.41	0

^a^Total fatty acids (TFA) (%); mean ± standard error for 3 replicates per diet (pooled whole tissues of 5 fish/replicate).

^b^Reference: no replacement of fish meal (FM) and fish oil (FO).

^c^Replacement of 33% of FM with *N. oculata* and 100% of FO with *Schizochytrium* sp.

^d^Replacement of 66% of FM with *N. oculata* and 100% of FO with *Schizochytrium* sp.

^e^Replacement of 100% of FM with *N. oculata* and 100% of FO with *Schizochytrium* sp.

^f,g,h^Mean values not sharing a superscript letter in the same row differ significantly (P < 0.05) from Tukey’s HSD test.

^i^Saturated fatty acids (SFA) is the sum of all fatty acids without double bonds.

^j^Monounsaturated fatty acids (MUFA) is the sum of all fatty acids with a single bond.

^k^Arachidonic acid (ARA).

^l^Omega-6 (n-6) Polyunsaturated fatty acids (PUFAs) (sum of all fatty acids with ≥ 2 double bonds (18:2, 18:3, 20:2, 20:3, 20:4, 22:4, 22:5).

^m^Alpha-linolenic acid (ALA).

^n^Eicosapentaenoic acid (EPA).

^o^Docosahexaenoic acid (DHA).

^p^Omega-3 (n-3) PUFAs (18:3, 18:4, 20:3, 20:4, 20:5, 22:5, 22:6).

^q^n-6 long-chain (LC) PUFA (20:2, 20:3, 20:4, 22:4, 22:5).

^r^n-3 LCPUFA(20:3, 20:4, 20:5, 22:5, 22:6).

^s^Ratio calculated for total n-3 PUFA: total n-6 PUFA (n-3/n-6).

### Amounts of major n-3 and n-6 PUFA (mg/g) in the fillet

**Figure 1:**
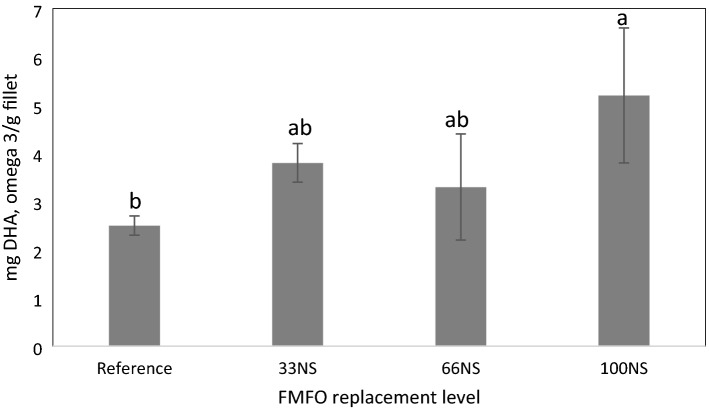
Docosahexaenoic acid (DHA), a key omega-3 fatty acid for human health, content in fish fillets fish fed the reference feed and three experiment diets. The experimental diets include a replacement of fishmeal (FM) with defatted biomass of *N. oculata* (N) to replace 33%, 66% or 100% of FM; and whole cell *Schizochytrium* sp. (S) to replace 100% of fish oil. Values are the mean of 3 replicates with pooled whole tissues of 5 fish per replicate. Values across the bars not sharing a common superscript were significantly different as determined by Tukey’s HSD test, P < 0.05. The error bars represent the standard error of the mean.

The amount of n-3 PUFAs, EPA and DHA did differ among diets (Supplementary Table S11). All diets that combined *Schizochytrium* with *N. oculata *defatted biomass enhanced the DHA deposition in the fillet. Tilapia fed fish-free feed, 100NS diet deposited a significantly higher (*p* < 0.05) amount of DHA (5.15 mg/g) than fish fed the reference diet which deposited DHA at 2.47 mg/g (Fig. 1). The EPA content of fish fed the reference diet was significantly higher (*p* < 0.05) compared to the other three diets and reflected the higher EPA supplied by this diet. The amounts of major n-6 PUFA deposition in the fish fillet (mg/g fillet) were not significantly different among diets.

### Degree of protein hydrolysis and in-vitro protein digestibility

We detected the highest degree of protein hydrolysis and in-vitro protein digestibility in the fish-free feed (100NS), although the difference was not statistically significant compared to the reference feed (Table 5).

**Table 5:** Degree of protein hydrolysis and of in vitro protein digestibility of experimental feeds.

	Reference^b^	33NS^c^	66NS^d^	100NS^e^
DH %^f^	4.29 ± 0.3^g,h^	3 ± 0.62^g,h^	2.25 ± 1.06^h^	5.64 ± 0.54^g^
IPD %^i^	85.3 ± 0.9^g,h^	80.8 ± 2.2^g,h^	78.1 ± 3.7^h^	87.7 ± 2.6^g^

^a^Mean ± standard error for 3 replicates per diet.

^b^Reference: no replacement of fish meal (FM) and fish oil (FO).

^c^Replacement of 33% of FM with *N. oculata* and 100% of FO with *Schizochytrium* sp.

^d^Replacement of 66% of FM with *N. oculata* and 100% of FO with *Schizochytrium* sp.

^e^Replacement of 100% of FM with *N. oculata* and 100% of FO with *Schizochytrium* sp.

^f^Degree of protein hydrolysis (DH).

^g,h^Mean values across the row not sharing a common superscript were significantly different P < 0.05 from Tukey’s HSD test.

^i^In-vitro protein digestibility (IPD)^43^.

### Economic analysis of fish-free feed formulated with microalgae blends

Here, we compared the estimated ingredient prices, the formulated feed prices and the ECR across experimental diets formulated with microalgae blends and the reference diet.

Results of the hedonic regression analysis show that the median price [and 95% confidence interval] is $0.44 [0.39, 0.49] and $2.38 [1.93, 2.57] per kg biomass for defatted *N. oculata* and whole cell *Schizochytrium* sp., respectively (Supplementary Table S5). While the median price of soybean meal is modestly greater (1.07 times) than the median price of defatted *N. oculata*, the median price of FM is nearly 3.5 times the median price of defatted *N. oculata*. In contrast to defatted *N. oculata* being much cheaper than FM, the median price of whole cell *Schizochytrium* sp. is roughly 1.4 times the median price of FO. Owing to this greater price of *Schizochytrium* sp. compared with FO, the median [and 95% confidence interval] price of the fish-free feed that combined defatted *N. oculata* meal with whole cell *Schizochytrium* sp. (100NS), at $0.68 [0.62, 0.73] per kg feed, was slightly greater than the reference diet at $0.64 [0.61, 0.68] per kg feed (Table 6).

**Table 6:** Formulated feed cost, feed conversion ratio, and economic conversion ratio of tilapia production.

Scenario	Formulated feed cost^a^	Feed conversion ratio^b^	Economic conversion ratio^a^
	($/kg feed)		($/kg tilapia)
Reference^c^	0.64 [0.61, 0.68]	1.61 ± 0.1	1.03 [0.95, 1.13]^d,e^
33NS^f^	0.72 [0.66, 0.78]	1.57 ± 0.1	1.14[1.00, 1.27]^e^
66NS^g^	0.70 [0.64, 0.76]	1.60 ± 0.1	1.12 [0.99, 1.25]^e^
100NS^h^	0.68 [0.62, 0.73]	1.40 ± 0.1	0.95 [0.82, 1.07]^d^
*F* value		3.00	13.49
*P* value		0.09	0.002

^a^Median [and 95% confidence interval].

^b^Mean ± standard error for 3 replicates per diet.

^c^Reference: no replacement of fish meal (FM) and fish oil (FO).

^d,e^Median values throughout the column not sharing a common superscript were significantly different as determined by Tukey’s HSD test, P < 0.05.

^f^Replacement of 33% of FM with *N. oculata* and 100% of FO with *Schizochytrium* sp.

^g^Replacement of 66% of FM with *N. oculata* and 100% of FO with *Schizochytrium* sp.

^h^Replacement of 100% of FM with *N. oculata* and 100% of FO with *Schizochytrium* sp.

**Figure 2:**
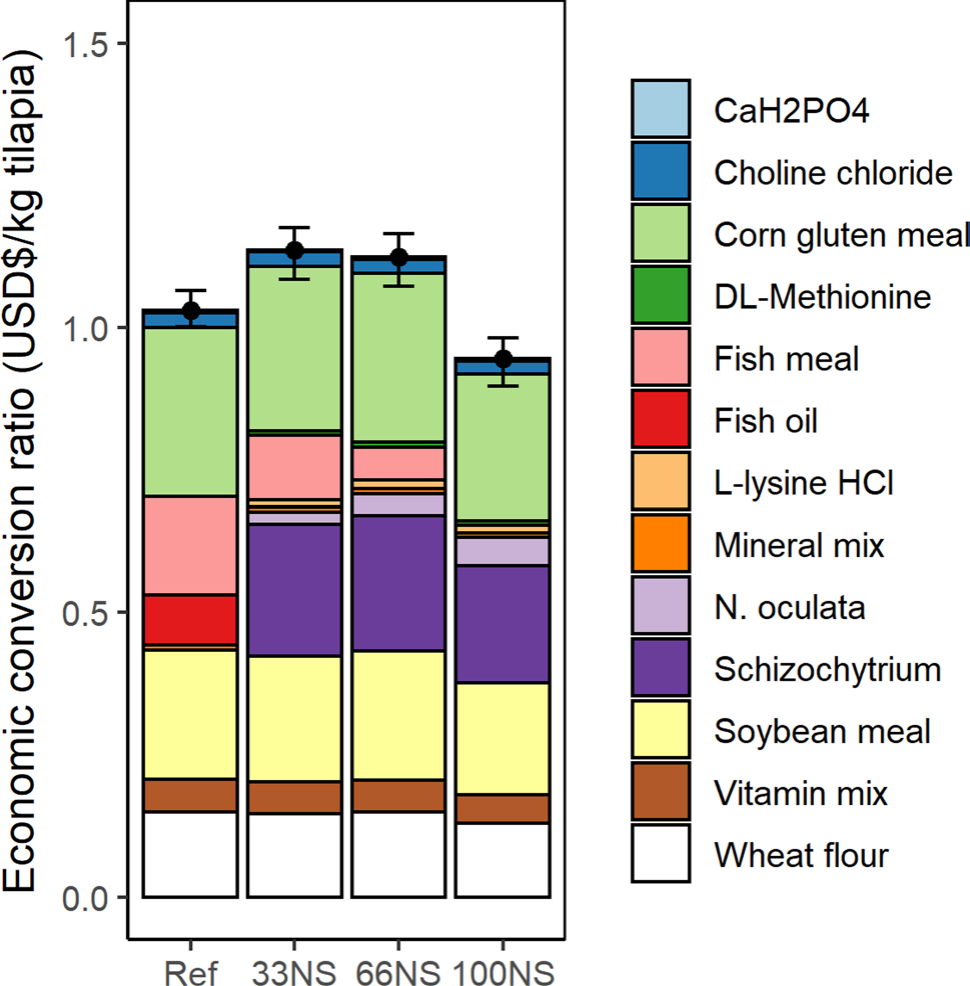
Economic conversion ratio of the reference (Ref) and experimental diets disaggregated by ingredient. The experimental diets include a replacement of fishmeal (FM) with defatted biomass of *N. oculata* (N) to replace 33%, 66% or 100% of FM; and whole cell *Schizochytrium* sp*.* (S) to replace 100% of fish oil. The error bars represent the 95% confidence interval.

The ECR, defined as the price of the formulated feed in US dollars per kg tilapia weight gain, of the fish-free feed was smaller than ECR of the reference diet (Fig. 2 and Table 6), despite the slightly greater price of the fish-free feed (100NS) compared to reference diet. We detected significant differences (*p* < 0.05) in ECR across all diets. While not significantly different, the ECR of the fish-free feed (100NS) at $0.95 [0.90, 0.98]/kg tilapia was roughly 92% the ECR of the reference diet ($1.03 [1.00, 1.07]/kg tilapia) (Fig. 2). This can be explained by the smaller FCR of the fish-free feed (1.40 ± 0.06) compared with reference diet (1.61 ± 0.05).

## Discussion

Our results demonstrate the feasibility of combining commercially available microalgal biomasses to formulate fish-free aquaculture feeds that are high-performing and show potential to become cost-competitive. This is the first report of successfully combining protein-rich-defatted biomass of one microalgal species with DHA-rich whole-cell biomass of another microalgal species to achieve full replacement of FM and FO ingredients in a tilapia feed formulation. This also is the first report of improved feed utilization metrics, including growth, weight gain, specific growth rate, and of beneficial DHA fatty acid profile in Nile tilapia fed a fish-free microalgal diet compared to a commercial feed formulation containing FM and FO. Production is increasing for both types of microalgal biomass used in the fish-free diet, indicating good potential to achieve economies of scale. Our estimate of the ECR for the fish-free diet supports the proposition that biomass from these microalgae will inevitably become cost competitive with FM and FO commodities.

### Nutritional benefit of combining *N. oculata* defatted biomass and *Schizochytrium* in the fish-free diet

The combination of *Schizochytrium* sp. and defatted biomass of *N. oculata* in the fish-free feed exhibited two major benefits. First, fish fed the fish-free feed had improved growth consistent with our prior observations that *Schizochytrium* sp. is a highly digestible ingredient for tilapia^33^ and that elevated levels of *Schizochytirum* sp. led to improved growth, FCR, and PER^30^. Second, we found the highest in-vitro protein digestibility in the fish-free feed, suggesting that protein originating from defatted *N. oculata* biomass was the most digestible when in the presence of highly digestible *Schizochytrium* sp., presumably due to the latter triggering certain digestive enzymes, release and activity. Thus, the combination of defatted *N. oculata* biomass and *Schizochytrium* sp. appears to be better suited to the digestive enzymes present in tilapia digestive systems than conventional diets with FMFO; and the presence of *Schizochytrium* sp. may support more efficient digestion of the fish free-feed at the higher inclusion levels of *N. oculata* defatted biomass. However, further research is necessary to elucidate the digestive enzyme profiles present under different dietary regimes and to assess the differences in the digestibility of microalgal fish-free feeds compared to conventional feed with FMFO.

Other studies also point to benefits of including *Schizochytrium* in aquafeeds. Our prior study reported better digestibility, improved growth, fillet protein, and lipid content by Nile tilapia fed diets with inclusion of *Schizochytrium* in fish-free feed. Similar results were reported in a study that found dietary inclusion of *Schizochytrium* sp. stimulated muscle or tissue development of Atlantic salmon^53^. Our observations of beneficial effects of including *Schizochytrium* in fish-free feed on the growth of tilapia is also consistent with findings in shrimp and barramundi, which demonstrated an algal derived DHA stimulated growth performance^54,55^. Moreover, high levels of a micronutrient, such as the carotenoid, astaxanthin, and bioactive compounds, in DHA-rich *Schizochytrium* could contribute to the growth of fish^30,55^.

Significantly lower weight gain of tilapia fed the reference feed compared to fish-free feed also seems consistent with the fact the FM and FO in reference diet had limited dietary 22:6n-3 DHA. This would cause increased energy expenditure for de novo DHA biosynthesis, given that DHA biosynthesis is a rather expensive metabolic exercise. Such diversion of energy to DHA biosynthesis would reduce the growth performance of tilapia.

The human health benefit of using highly digestible 22:6n-3 DHA-rich *Schizochytrium* is reflected in this study, given that tilapia fed the fish-free feed yielded the highest amount of 22:6n-3 DHA in fillet—almost twice that of conventional feed (Supplementary Table S6). Results are consistent with our previous findings where increasing levels of *Schizochytrium *sp. corresponded to reduced levels of FO in tilapia feed and resulted in significant increases in fillet 22:6n-3 DHA deposition compared to a reference diet containing FMFO^30^.

Nile tilapia is not an oily fish like salmon, but nevertheless deserves efforts to improve nutritional value of farmed fish because it is produced in huge tonnages and is an important component of human diets in many parts of the world, especially Asia and Africa. Thus, improvement of tilapia nutritional value through increased levels of DHA could benefit a very large number of people, many of whom have low levels of n-3 LC-PUFA in their diets^23^. Our results support the relative ease of enhancing the n-3 LC PUFA composition of tilapia fillets, while also achieving a fish-free diet, by combining *Schizochytrium *sp. and *N. oculata* defatted biomass. Tilapia with elevated DHA levels after eating fish-free feed will have tremendous market potential^56^. Feed manufacturers can exploit this feature to market aquafeeds to aquaculturists aiming to cater to health-conscious consumers who are willing to pay a premium for DHA-enhanced tilapia fillets. Tilapia fed reference feed exhibited significantly increased amounts of 20:5n-3 EPA compared to microalgae-inclusion diets due to a higher concentration of 20:5n-3 EPA in the reference diet. Our results on fillet deposition of ALA, EPA and DHA can be explained by prior research and the relative abundance of these fatty acids in *Schizochytrium *sp.

### Impacts of fish-free diet on macrominerals and trace elements

The literature has little data on the elemental composition of microalgae; and we found that most of the essential macrominerals and trace elements were at higher levels in *N. oculata* defatted biomass and *Scizochytrium* sp whole cells (Table 3) than in conventional terrestrial feed ingredients^57^. We found higher levels for most macrominerals in the *Scizochytrium* sp whole cells than *N. oculata* defatted biomass, and higher levels of trace elements in the *N. oculata* defatted biomass than in *Scizochytrium* sp whole cells (Table 3). Depositions of macrominerals and several trace elements in tilapia fillet were not significantly different among all dietary treatments (Table 3). We found non-detectable levels of boron, mercury, and lead in tilapia fillets across all diets. Moreover, most of the trace element concentration in fillet was lower than the concentration of all experimental diets. We previously suggested that these trace elements may be excreted and less absorbed by Nile tilapia^58,59^. We detected the lowest level (0.03 mg kg^−1^) of total arsenic in the fish-free microalgae feeds and the highest level (0.33 mg kg^−1^) in reference feed (Supplementary Table S7). However, the level of total arsenic in all the diets (0.03–0.33 mg kg^−1^) including reference feed was below the European Union level of 10 mg kg^−1^ set for in aquaculture feed^60^. High levels of arsenic have been previously reported in FOs, thus contributing considerably to higher arsenic levels in commercial aquaculture feeds^61–63^. The level of total arsenic in the fillet of tilapia did not differ across the diets (Table 3), and the levels were in the range between 0.14-0.21 mg kg^−1^ lower than reported values in Atlantic salmon fillet (0.3–1.1 mg kg^−1^)^64^.

### Feed conversion ratio (FCR) considerations

FCR is a key driver of farming efficiency, economic and environmental performance. Improving the FCR of farmed tilapia through improved feed technology would help increase the cost effectiveness of fish-free diets. Tilapia farming can further reduce the FCR close to 1:1 by a variety of means including better feed formulations using highly digestible feed ingredients, use of appropriate pellet size for each life stage, and better on-farm feed management practices (e.g., storage and feeding rates). Extruded sinking pelleted feed could improve overall FCR; moreover, extrusion or enzymatic processing of under-utilized, defatted biomass of microalgae, such as *N. oculata* used in this study, could further improve the FCR of fish-free feed, and also help push feed formulated with microalgae towards being cost-competitive with conventional feed^17,65^.

### Economic analysis of fish-free feed formulated with microalgae blends

Our estimate of the market price of defatted *N. oculata* meal is in good agreement with another study that used hedonic methods to estimate the of market price of defatted *N. oculata* meal^45^. However, key differences between our study and the study conducted by Maisashvili et al. is that we used more recent commodity prices (January 2010 to December 2019 instead of January 2005 to December 2012) and the list of commodities used in our analysis are more representative of tilapia feed ingredients instead of ingredients for carnivorous fish and shrimp feed. With respect to whole cell *Schizochytrium* sp., we are unaware of other studies using hedonic methods to estimate the implied market price of this ingredient. Nevertheless, our implied price results for whole cell *Schizochytrium* sp. are in general agreement with studies that have used alternative methods^66,67^.

The similar estimated costs of the fish-free feed (100NS) and reference diet suggest that using combinations of microalgal biomass, that are on track to achieve economies of scale, is a feasible strategy for achieving large-scale production of cost-competitive fish-free diets. An emerging path to economies of scale for the two microalgae used in this study is a biorefinery business model whereby oil rich fractions of the microalgal biomass are marketed as high-value products, such as omega-3 rich human supplements, and other fractions as lower-priced feed ingredients^68,69^. *N. oculata* contains an appreciable amount of the omega-3 fatty acid, EPA^70^. The projected global growth of over 14% in omega-3 fatty acids from microalgae in the near future will result in a large supply of defatted biomass^67^. Furthermore, the production of *Schizochytrium* sp., already at commercial-scale, is also anticipated to grow, as the projected compound annual growth rate of DHA from microalgae sources is expected to exceed 10% in the near future^67^.

In order for such high-performing fish-free feed for tilapia to succeed in the market, we acknowledge that *Schizochytrium* sp. needs to become cost-competitive with FO sources for aquaculture feeds. Analysts predict ongoing technological improvements and R&D efforts to produce *Schizochytrium* sp. will quickly make it a cost competitive substitute for FO due to lower production costs and higher market availability^71,72^. FO substitutes with *Schizochytrium* sp have emerged within the last year with new products from many agribusiness giants and animal nutrition companies (Corbion, BioMar, Archer Daniels Midland and Veramaris), presumably due to favorable economics and high production volumes. A commercial producer of *Schizochytrium *oil, Veramis, recently joined a global challenge to sell the most “fish-free” oil for aquafeed to reduce demand pressures on wild-caught stocks, the fish-free feed (F3) FO Challenge^73^. Alternative feed ingredients like natural marine algal oil have also recently been approved for use in the supply chain by the UK retailer, Tesco^74^. Given the proliferation of alternative feed ingredients by global industry leaders and stakeholders (aquafeed company, innovators, aquafarmers, investors, and aquaculture supply chain), market opportunities appear to be growing and evolving for using microalgal protein and oil for fish-free feed^75,76^.

## Conclusion

Our results provide a framework for the development of fish-free feeds and the first evidence of a high performing feed for tilapia that combines two different marine microalgae. Defatted marine microalgae, a protein-rich biomass left over after extracting oil for other products, is currently under-utilized (often creating disposal problems even though it is food-grade), and is increasingly available as the algal-oil nutraceutical market grows. Advancing the use of microalgal defatted biomass in aquafeeds would improve the sustainability of aquaculture by reducing its reliance on FM extracted from forage fisheries. Combining under-utilized defatted biomass protein with DHA-rich marine microalga in the fish-free feed resulted in better tilapia growth compared with fish fed a conventional diet containing FMFO. Furthermore, tilapia fed the fish-free feed yielded the highest amount of DHA in the fillet, almost twice higher than in those fed conventional feed. Thus, feeding a DHA-rich, microalgae blended diet to farmed tilapia is a practical way to improve human health benefits of eating farmed tilapia. Moreover, these results suggest other kinds of microalgae combinations are possible and worthy of future investigation. Our fish-free formulation also shows potential cost-competitiveness, given that the ECR of the fish-free diet was slightly lower, though not significantly different, than the reference diet. The microalgal ingredients in our fish-free feed, thus, show potential to supply the expanding aquaculture industry with a stable and affordable supply of healthy protein and oil for fish-free feed, doing so without causing harm to oceans or food security of resource-poor people.

## Supplementary information

Supplementary Information.
