# Supplementary material for: Microalgae-blend tilapia feed eliminates fishmeal and fish oil, improves growth, and is cost viable
Source: Sci Rep. 2020 Nov 12;10:19328. doi: 10.1038/s41598-020-75289-x (PMC7665073; doi:10.1038/s41598-020-75289-x)
Supplement: Supplementary file 1 — Supplementary Information. [file 41598_2020_75289_MOESM1_ESM.docx]

Supplementary Information Appendix

**Microalgae-blend tilapia feed eliminates fishmeal and fish oil, improves growth, and is cost viable**

Pallab K. Sarker^a*^**,** Anne R. Kapuscinski^a^, Brandi McKuin^a^, Devin S. Fitzgerald^a^, Hannah M. Nash^b^, Connor Greenwood^a^

^a^Current address: Environmental Studies Department, University of California Santa Cruz, Santa Cruz, CA 95060, USA

^b^Current address: Health Professions Program, Sciences, Mathematics and Biotechnology, UC Berkeley Extension, 1995 University Ave., Suite 200, Berkley, CA 94704-7000, USA

**Supplementary Methods**

*Freight model*

For U.S. commodities, we used data from the USDA Marketing Service and the USDA Economic Research Service to inform our selection of the origin city. To inform our selection of the destination city, we selected a U.S. agricultural port in close proximity to the origin city^1^. We applied average freight revenues to the distances for each transit mode to estimate the domestic shipping costs^2^. We applied the producer’s price index for crude oil to the shipping cost for the year 2018 to estimate the shipping cost for the year 2019.

We estimated the average annual international shipping costs using bulk grain ocean freight rates^3^. We used the bulk grain ocean freight shipping rate from the U.S. Gulf to Japan. To estimate the cost on a ton-km basis, we assumed the origin port was the Port of New Orleans and the destination port was the Port of Tokyo.

*Assessment of model fit*

To assess the fit of the models we used seven criteria: 1) test significance of random effects; 2) the coefficient of determination; 3) test for linearity; 4) homogeneity of variance; 5) identify influential observations; 6) test that the residuals are normally distributed; and 7) validate the model with commodity prices.

To test the significance of the mixed-effects model compared to a linear model with fixed-effects only, we compared the Akaike information criterion (AIC) of the linear model with only fixed effects to the model that included both fixed effects and random-effects using ANOVA (Dunn and Smyth, 2018).

We calculated the coefficient of determination of the models in R^4,5^.

We tested linearity, graphically. We plotted the model residuals (the difference between the observed value and the model-estimated value) versus the predictors and assessed that the model meets the criteria if we did not see a pattern emerge (i.e. anything that looks non-random)^6-8^.

To test the homogeneity of variance, we used the Levene or the Breusch-Pagan test to confirm that the variance of the residuals is equal across groups^6,7^.

We identified influential observations using the criteria provided in Dunn and Smyth (2018)^8^. We used the influence measures function in R to identify observations that significantly influence the model fit.

We tested that the residuals are normally distributed, graphically with a QQ plot^7,8^. We used QQ plots to estimate where the standardized residuals lie with respect to normal quantiles and visually inspected whether we detected strong deviation from the provided line.

Following Bryant et al., 2012^9^, we validated the model with commodity price data that we used in the hedonic regression. In the case of defatted *N. oculata*, we plotted the results of the predicted values versus the actual values of eight different meal commodities (canola meal, corn gluten meal, cotton seed meal, fish meal, linseed meal, palm kernel meal, soybean meal, and sunflower meal) and evaluated the coefficient of determination. In the case of *Schizochytrium* sp. oil, we used the six different oil commodities (canola/rapeseed oil, corn oil, fish oil, palm oil, soybean oil, and sunflower oil).

**Defatted *N. oculata* meal model fit analysis**

The random effects of the mixed-model were significant according to (p-value 9.584e-06 with 6 degrees of freedom). The AIC value of the mixed-effect model was 1413.9 compared with 1435.1 for the fixed-effects only. The coefficient of determination of the mixed-effect model was 0.995262. The plot of the model residuals (the difference between the observed value and the model-estimated value) versus the predictor is presented in Figure S2. The results of the Levene test is presented in Table S7. Although we did identify influential observations, when we ran the model without the highly influential observations, the coefficient of determination did not improve. Thus, we did not omit these observations from the model. The plot of the normal QQ plots is presented in Figure S3. The plot of the model validation (actual versus predicted values) is presented in Fig. S4 with a coefficient of determination of 0.9952.

***Schizochytrium* sp. oil model fit analysis**

The random effects of the mixed-model were significant according to (p-value 2.899e-16 with 9 degrees of freedom). The AIC value of the mixed-effect model was 886.21

compared with 961.94 for the fixed-effects only. The coefficient of determination of the mixed-effect model was 0.980565. The plot of the model residuals (the difference between the observed value and the model-estimated value) versus the predictor is presented in Figure S5. The results of the Levene test is presented in Table S10. However, because the p-values of β_2_ (14:0^2^) and β_4_ (14:0) were < 0.05, we used the Breusch-Pagan test for heteroskedacity. The result of the Breusch-Pagan test resulted in a p-value of 0.2867. Although we did identify influential observations, when we ran the model without the highly influential observations, the coefficient of determination did not improve. Thus, we did not omit these observations from the model. The plot of the normal QQ plots is presented in Figure S6. The plot of the model validation (actual versus predicted values) is presented in Fig. S7 with a coefficient of determination of 0.9831.

**Table S1.** Estimation of percent of global fishmeal and fish oil production used in manufactured tilapia feeds in 2017.

| Global catch to non-food/ FMFO (t) ^a^ | FM yield (%) ^b^ | FO yield (%) ^b^ | FMFO yield (%) ^c^ | FMFO yield (t) ^d^ | Global tilapia FMFO (t) use ^e^ | Global FMFO (%) for tilapia feed ^f^ |
| --- | --- | --- | --- | --- | --- | --- |
| 19,000,000 | 22.5 | 5 | 27.5 | 5,225,000 | 937,992 | 17.95 |

^a^ Assumed all non-food wild catch to make fishmeal and fish oil (FMFO)^10^

^b^ One tonne (t) of feed grade wild fish is assumed to yield 225 kg of fishmeal (22.5%) and 50 kg of fish oil (5%) respectively^11^.

^c^ FMFO yield (%)=(FM yield %+ FO yield%).

^d^ FMFO yield (t)= Global catch to non-food/ FMFO (t)* FMFO yield (%).

^e^ Global tilapia FMFO (t) use from Tacon (2019)^12^.

^f^ Global FMFO (%) for tilapia feed= (global tilapia FMFO (t) use/FMFO yield)*100.

**Table S2.** Whole body proximate composition (wet weight basis) of Nile tilapia after 184 days on the experimental diets.

|  |  | Whole body ^a^ |  |  | ANOVA |  |
| --- | --- | --- | --- | --- | --- | --- |
|  | Reference ^b^ | 33NS ^c^ | 66NS ^d^ | 100NS ^e^ | *F* Value | *P* value |
| Moisture | 78.18 ± 0.17 | 74.42 ± 2.47 | 73.7 ± 3.38 | 70.94 ± 2.2 | 1.59 | 0.26 |
| Protein | 13.28 ± 0.81 | 15.3 ± 1.37 | 15.67 ± 2.6 | 17.48 ± 0.97 | 1.16 | 0.38 |
| Fat | 1.7 ± 0.56 | 3.28 ± 1.35 | 3.22 ± 0.43 | 5.07 ± 0.87 | 2.48 | 1.3 |
| Fiber | 0.06 ± 0.03 | 0.06 ± 0.01 | 0.04 ± 0.01 | 0.05 ± 0.02 | 0.07 | 0.97 |
| Ash | 4.55 ± 0.72 | 5.09 ± 0.43 | 4.6 ± 0.76 | 4.56 ± 0.32 | 0.19 | 0.89 |

^a^ Mean ± Standard Error (n=3 replicates per diet; pooled whole tissues of 5 fish/replicate). No significant diet differences were detected (P>0.05) for whole body proximate compositions.

^b^ Reference: no replacement of fish meal (FM) and fish oil (FO).

^c^ Replacement of 33% of FM with *N. oculata* and 100% of FO with *Schizochytrium* sp.

^d^ Replacement of 66% of FM with *N. oculata* and 100% of FO with *Schizochytrium* sp.

^e^ Replacement of 100% of FM with *N. oculata* and 100% of FO with *Schizochytrium* sp.

**Table S3**. Proximate fillet composition (wet weight basis) of Nile tilapia fed experimental diets for 184 days.

| Proximate composition (%) | |  |  |  |  |  |
| --- | --- | --- | --- | --- | --- | --- |
|  | | Fillet ^a^ |  |  | ANOVA |  |
|  | Reference ^b^ | 33NS ^c^ | 66NS ^d^ | 100NS ^e^ | *F* Value | *P* Value |
| Protein | 18.38 ± 0.56 | 19.43 ± 0.91 | 22.91 ± 3.26 | 24.48 ± 2.28 | 1.94 | 0.2 |
| Fat | 0.84 ± 0.01^f^ | 0.88 ± 0.03^f^ | 0.92 ± 0.14^f^ | 1.85 ± 0.26^g^ | 11.02 | 0.003 |
| Fiber | 0.07 ± 0.02 | 0.07 ± 0.02 | 0.07 ± 0 | 0.08 ± 0.01 | 0.11 | 0.95 |
| Ash | 1.6 ± 0.14 | 1.56 ± 0.01 | 1.76 ± 0.31 | 1.88 ± 0.23 | 0.5 | 0.69 |

^a^ Mean ± Standard Error (n=3 replicates per diet; pooled whole tissues of 5 fish/replicate).

^b^ Reference: no replacement of fish meal (FM) and fish oil (FO).

^c^ Replacement of 33% of FM with *N. oculata* and 100% of FO with *Schizochytrium* sp.

^d^ Replacement of 66% of FM with *N. oculata* and 100% of FO with *Schizochytrium* sp.

^e^ Replacement of 100% of FM with *N. oculata* and 100% of FO with *Schizochytrium* sp.

^f,g^ Values across the row not sharing a common superscript were significantly different as determined by Tukey’s HSD test, P<0.05.

**Table S4**. Essential amino acid content (wet weight basis) of fillets from Nile tilapia after 184 days on the experimental diets.

| Essential amino acids |  |  | Fillet ^a^ |  | ANOVA |  |
| --- | --- | --- | --- | --- | --- | --- |
|  | Reference ^b^ | 33NS ^c^ | 66NS ^d^ | 100NS ^e^ | *F* Value | *P* Value |
| Methionine | 1.93 ± 0.14^f^ | 1.39 ± 0.18^g^ | 2.01 ± 0.07^f^ | 1.97 ± 0.08^f^ | 5.37 | 0.02 |
| Lysine | 6.08 ± 0.6 | 4.04 ± 0.56 | 6.18 ± 0.43 | 5.96 ± 0.36 | 4.19 | 0.06 |
| Phenylalanine | 2.67 ± 0.23 | 1.87 ± 0.27 | 2.74 ± 0.12 | 2.68 ± 0.16 | 4.25 | 0.06 |
| Leucine | 5.3 ± 0.46 | 3.62 ± 0.53 | 5.38 ± 0.29 | 5.11 ± 0.26 | 4.24 | 0.06 |
| Isoleucine | 2.61 ± 0.41 | 1.56 ± 0.17 | 2.58 ± 0.4 | 2.3 ± 0.25 | 2.26 | 0.15 |
| Threonine | 3.24 ± 0.28 | 2.18 ± 0.3 | 3.26 ± 0.2 | 3.16 ± 0.22 | 4.35 | 0.06 |
| Valine | 2.98 ± 0.42 | 1.84 ± 0.22 | 2.94 ± 0.37 | 2.74 ± 0.25 | 2.73 | 0.11 |
| Histidine | 1.55 ± 0.19^f^ | 1.06 ± 0.14^g^ | 1.76 ± 0.09^f^ | 1.56 ± 0.13^f^ | 4.26 | 0.04 |
| Arginine | 4.77 ± 0.29 | 3.36 ± 0.47 | 4.8 ± 0.16 | 4.62 ± 0.3 | 4.56 | 0.038 |

^a^ Mean ± Standard Error (n=3 replicates per diet; pooled whole tissues of 5 fish/replicate).

^b^ Reference: no replacement of fish meal (FM) and fish oil (FO).

^c^ Replacement of 33% of FM with *N. oculata* and 100% of FO with *Schizochytrium* sp.

^d^ Replacement of 66% of FM with *N. oculata* and 100% of FO with *Schizochytrium* sp.

^e^ Replacement of 100% of FM with *N. oculata* and 100% of FO with *Schizochytrium* sp.

^f,g^ Values across the row not sharing a common superscript were significantly different as determined by Tukey’s HSD test, P<0.05. ND, not detectable (<0.000 ug/g).

**Table S5.** Ingredient prices.

| Ingredient | Price ^a^ | Source |
| --- | --- | --- |
|  | ($ / kg) |  |
| Fish meal (n=30) | 1.54 [1.45, 1.63] | ^13^ |
| *N. oculata* co-product (n=30) | 0.44 [0.40, 0.49] | This study |
| Fish oil (n=30) | 1.68 [1.64, 1.76] | ^13^ |
| *Schyzochytrium* sp. (n=30) | 2.38 [1.93, 2.57] | This study |
| Corn gluten meal (n=30) ^b^ | 0.62 [0.59, 0.66] | ^14^ |
| Soybean meal (n=30) ^b,c^ | 0.47 [0.45, 0.50] | ^14^ |
| Wheat flour (n=30) | 0.47 [0.44, 0.51] | ^15^ |
| Dicalcium Phosphate (21%) (n=8) ^d,e^ | 0.36 [0.30, 0.41] | ^16^ |
| L-lysine HCl (n=7) ^f^ | 1.60 [1.40, 1.80] | ^16^ |
| DL-Methionine (n=6) ^g^ | 2.65 [1.60, 4.50] | ^16^ |
| Choline chloride (n=7) ^h^ | 0.80 [0.38, 0.90] | ^16^ |
| Mineral mix (n=1) ^i^ | 0.56 | Sigma Aldrich |
| Vitamin mix (n=1) ^i^ | 3.54 | Sigma Aldrich |

^a^ Median [and 95% confidence interval] calculated from bootstrap analysis.

^b^ Custom report: "Livestock", "Feedstuffs".

^c^ 49.7% protein content.

^d^ Search term: “Di-calcium phosphate DCP”.

^e^ Search narrowed to verified suppliers that produce more than 1Mt of product and have made a sale six months prior to Nov. 4

2019.

^f^ Search term: “Feed Grade Lysine”.

^g^ Search term: “Feed Grade DL-methionine”.

^h^ Search term: “Choline Chloride 60%”.

^i^ Vitamin and Mineral mixes created in lab.

**Table S6.** Amounts of lipid and major omega-3 (n-3) and n-6 polyunsaturated fatty acid (PUFA) in the fillet (wet weight basis) of Nile tilapia fed experimental diets for 184 days.

|  |  | Fillet PUFA (mg/g) ^a^ | |  | ANOVA |  |
| --- | --- | --- | --- | --- | --- | --- |
| Fatty acid (mg/g fillet) | Reference ^b^ | 33NS ^c^ | 66NS ^d^ | 100NS ^e^ | *F* Value | *P* Value |
| 18:2n-6 LA ^f^ | 4.61 ± 0.1 | 3.69 ± 0.39 | 3.66 ± 0.61 | 5.67 ± 0.8 |  |  |
| 20:4n-6 ARA ^g^ | 1.03 ± 0.07 | 0.69 ± 0.07 | 0.78 ± 0.1 | 0.81 ± 0.15 | 2.62 | 0.12 |
| 18:3n-3 ALA ^h^ | 0.25 ± 0.02 | 0.2 ± 0.02 | 0.17 ± 0.03 | 0.29 ± 0.06 | 3.54 | 0 |
| 20:5n-3 EPA ^i^ | 0.51 ± 0.08^j^ | 0.13 ± 0.02^k^ | 0.14 ± 0.01^k^ | 0.17 ± 0.05^k^ | 13.63 | 0.02 |
| 22:6n-3 DHA ^l^ | 2.47 ± 0.19^k^ | 3.88 ± 0.41^jk^ | 3.32 ± 1.17^jk^ | 5.15 ± 1.42^j^ | 36.45 | 0 |

^a^ Mean ± Standard Error (n=3 replicates per diet; pooled whole tissues of 5 fish/replicate).

^b^ Reference: no replacement of fish meal (FM) and fish oil (FO).

^c^ Replacement of 33% of FM with *N. oculata* and 100% of FO with *Schizochytrium* sp.

^d^ Replacement of 66% of FM with *N. oculata* and 100% of FO with *Schizochytrium* sp.

^e^ Replacement of 100% of FM with *N. oculata* and 100% of FO with *Schizochytrium* sp.

^f^ Linoleic Acid (LA).

^g^ Arachidonic acid (ARA).

^h^ Alpha linolenic acid (ALA).

^i^ Eicosapentaenoic acid (EPA).

^j,k^ Mean values across the row not sharing a common superscript were significantly different as determined by Tukey’s HSD test, P<0.05.

^l^ Docosahexaenoic acid (DHA).

**Table S7**. Macro minerals and trace elements in the experimental diets.

|  |  | Diet |  |  |
| --- | --- | --- | --- | --- |
|  | Reference ^a^ | 33NS ^b^ | 66NS ^c^ | 100NS ^d^ |
| *Macro minerals (%)* | |  |  |  |
| Phosphorus | 7.56 | 8.75 | 9.47 | 7.74 |
| Calcium | 6.46 | 7.05 | 5.41 | 4.08 |
| Magnesium | 1.43 | 1.59 | 2.35 | 1.91 |
| Potassium | 9.6 | 10.2 | 14.83 | 11.06 |
| Sulfur | 4.58 | 6.65 | 8.0 | 6.97 |
| *Trace elements (mg kg^−1^)* | |  |  |  |
| Copper | 9.57 | 11.46 | 17.52 | 16.69 |
| Iron | 78.23 | 101.48 | 145.85 | 119.31 |
| Manganese | 18.69 | 23.71 | 33.66 | 24.3 |
| Selenium | 0.33 | 0.32 | 0.39 | 0.44 |
| Zinc | 38.73 | 40.7 | 50.07 | 36.72 |
| Arsenic | 0.33 | 0.04 | 0.03 | 0.03 |
| Boron | 15.01 | 14.95 | 23.32 | 16.93 |
| Aluminum | 15.68 | 24.86 | 45.63 | 42.46 |
| Mercury | ND ^e^ | ND | ND | ND |
| Lead | ND | ND | ND | ND |
| Molybdenum | ND | ND | ND | ND |

^a^ Reference: no replacement of fish meal (FM) and fish oil (FO).

^b^ Replacement of 33% of FM with *N. oculata* and 100% of FO with *Schizochytrium* sp.

^c^ Replacement of 66% of FM with *N. oculata* and 100% of FO with *Schizochytrium* sp.

^d^ Replacement of 100% of FM with *N. oculata* and 100% of FO with *Schizochytrium* sp.

^e^ Not detectable (ND) (<0.000 ug/g).

**Table S8**. Proximate chemical composition, gross energy, essential amino acid and fatty acid profiles of the *Schizochytrium* sp. and *N. oculata* defatted biomass as test ingredients.

|  |  | Ingredients |
| --- | --- | --- |
|  | *Schizochytrium* sp. | *N. oculata* defatted biomass |
| *Proximate composition (g kg^-1^as is)* |  |  |
| Crude protein | 119 | 497 |
| Lipid | 541 | 48 |
| Ash | 87 | 86 |
| Fibre | 2.4 | 3.7 |
| Energy, kJ g^-1^ | 17.7 | 1.3 |
| *Essential amino acids (g kg^-1^ in the weight of ingredient as is)* | | |
| Arginine | 0.8 | 26.0 |
| Lysine | 0.5 | 27.0 |
| Isoleucine | 0.4 | 21.0 |
| Leucine | 0.7 | 42.0 |
| Histidine | 0.3 | 9.0 |
| Methionine | 1.2 | 10.0 |
| Phenylalanine | 0.4 | 24.3 |
| Threonine | 0.4 | 23.6 |
| Tryptophan | 0.2 | 4.5 |
| Valine | 0.6 | 29.0 |

**Table S9.** Fatty acid content (% of total fatty acids) test ingredients whole cell dried *Schizochytrium* sp. and *N. oculata* defatted biomass used in the experimental diets.

| Fatty acids  (% of TFA) | *Schizochytrium* sp. | *N. oculata* defatted biomass |
| --- | --- | --- |
| 14:00 | 9.3 | 5.09 |
| 15:00 | 0.5 | 0.31 |
| 16:00 | 24.4 | 21.31 |
| 17:00 | ND ^a^ | 0.15 |
| 18:00 | 0.5 | 0.44 |
| 20:00 | 0.1 | 0.13 |
| 22:00 | 0.1 | ND |
| 24:00 | ND | ND |
| 16:1n-9 | ND | 6.32 |
| 16:1n-7 | 0.2 | 23.53 |
| 18:1n-9 | 0.1 | 3.28 |
| 18:1n-7 | ND | 0.83 |
| 20:1n-9 | ND | ND |
| 20:1n-7 | ND | ND |
| 22:1n-11 | ND | ND |
| 22:1n-9 | ND | ND |
| 24:1n-9 | 1.4 | ND |
| 18:2n-6 | ND | 2.02 |
| 18:3n-6 | 0.2 | 0.46 |
| 20:2n-6 | ND | ND |
| 20:3n-6 | 0.3 | 0.40 |
| 20:4n-6 ARA ^b^ | 1.4 | 5.82 |
| 22:4n-6 | 0.1 | ND |
| 22:5n-6 | 15.8 | ND |
| 18:3n-3 ALA ^c^ | ND | 0.08 |
| 18:4n-3 | 0.6 | 0.00 |
| 20:3n-3 | 0.1 | ND |
| 20:4n-3 | 0.8 | ND |
| 20:5n-3 EPA ^d^ | 0.8 | 27.94 |
| 22:5n-3 | 0.4 | ND |
| 22:6n-3 DHA ^e^ | 43.2 | ND |
| Total SFA ^f^ | 9.2 | 28.53 |
| Total MUFA ^g^ | 1.7 | 34.34 |
| Total n-3 PUFA ^h^ | 45.9 | 28.03 |
| Total n-6 PUFA ^i^ | 17.8 | 8.71 |
| Total PUFA ^j^ | 63.7 | 37.13 |
| Total n-6 LCPUFA ^k^ | 17.6 | 6.22 |
| Total n-3 LCPUFA ^l^ | 45.3 | 27.94 |

^a^ Not detectable (ND) (<0.000 ug/g).

^b^ Arachidonic acid (ARA).

^c^ Alpha linolenic acid (ALA).

^d^ Eicosapentaenoic acid (EPA).

^e^ Docosahexaenoic acid (DHA).

^f^ Saturated fatty acids (SFA) is the sum of all fatty acids without double bonds.

^g^ Monounsaturated fatty acids (MFA) is the sum of all fatty acids with a single bond.

^h^ Polyunsaturated fatty acids (PUFA) is the sum of all fatty acids with ≥2 double bonds.

^i^ Omega-3 (n-3) PUFA (18:3, 18:4, 20:3, 20:4, 20:5, 22:5, 22:6).

^j^ n-6 PUFA (sum of all fatty acids with ≥2 double bonds (18:2, 18:3, 20:2, 20:3, 20:4, 22:4, 22:5).

^k^ n-6 long-chain (LC) PUFA (20:2, 20:3, 20:4, 22:4, 22:5).

^l^ n-3 LCPUFA (20:3, 20:4, 20:5, 22:5, 22:6).

**Table S10.** Macro minerals and trace elements in the *Schizochytrium* sp.

and *N. oculata* defatted biomass.

|  | Ingredients | |
| --- | --- | --- |
|  | *Schizochytrium* sp | *N. oculata* defatted biomass |
| *Macro minerals (%)* | |  |
| Phosphorus | 8.44 | 1.4 ±0.0 |
| Calcium | 1.03 | 0.8 ± 0.0 |
| Magnesium | 5.77 | 0.6 ± 0.0 |
| Potassium | 1.13 | 1.0 ± 0.0 |
| Sulfur | 21.63 | 0.5 ± 0.0 |
| *Trace elements (mg kg^−1^)* | |  |
| Copper | 2.75 | 61.0 ± 3.0 |
| Iron | 10.32 | 659.8 ± 14.9 |
| Manganese | 4.4 | 91.6 ± 2.2 |
| Selenium | ND ^a^ | 1.0 ± 0.0 |
| Zinc | 3.85 | 47.0 ± 1.8 |
| Boron | 3.22 | 1.0 ± 0.0 |
| Aluminum | BDL ^b^ | 468.5 ± 53.7 |
| Molybdenum | 0.11 | 0.4 ± 0.1 |
| Arsenic | 0.03 | 0.2 ± 0.0 |
| Mercury | ND | ND |
| Lead | ND | 1.1 ± 0.1 |

^a^ Not detectable (ND) (<0.000 ug/g).

^b^ Beyond detectable limits (BDL).

**Table S11.** Fatty acid (% of total fatty acids) content of the experimental diets

| Diet TFA % | Reference ^a^ | 33NS ^b^ | 66NS ^c^ | 100NS ^d^ |
| --- | --- | --- | --- | --- |
| 14:00 | 4.01 | 4.49 | 4.47 | 4.33 |
| 15:00 | 0.34 | 0.36 | 0.36 | 0.33 |
| 16:00 | 19.78 | 21.83 | 21.23 | 21.37 |
| 17:00 | 0.42 | 0.33 | 0.35 | 0.39 |
| 18:00 | 5.11 | 3.8 | 3.76 | 3.33 |
| 20:00 | 0.26 | 0.3 | 0.28 | 0.27 |
| 22:00 | 0.19 | 0.18 | 0.22 | 0.22 |
| 24:00 | 0.21 | 0.27 | 0.31 | 0.3 |
| Total SFA ^e^ | 30.33 | 31.55 | 30.97 | 30.53 |
| 16:1n-9 | 0.19 | 0.36 | 0.41 | 0.62 |
| 16:1n-7 | 5.52 | 1.39 | 1.61 | 1.9 |
| 18:1n-9 | 22.42 | 18.36 | 16.51 | 13.62 |
| 18:1n-7 | 2.71 | 1.38 | 1.33 | 1.21 |
| 20:1n-9 | 0.77 | 0.32 | 0.22 | 0.2 |
| 20:1n-7 | 0.08 | 0 | 0 | 0 |
| 22:1n-11 | 0.08 | 0 | 0 | 0 |
| 22:1n-9 | 0.19 | 0.34 | 0.22 | 0 |
| 24:1n-9 | 0 | 0 | 0 | 0 |
| Total MUFA ^f^ | 31.98 | 22.15 | 20.31 | 17.55 |
| 18:2n-6 | 23.2 | 22.29 | 23.28 | 22.57 |
| 18:3n-6 | 0.14 | 0.11 | 0.12 | 0.13 |
| 20:2n-6 | 0.23 | 0.16 | 0.11 | 0.08 |
| 20:3n-6 | 0.21 | 0.24 | 0.28 | 0.2 |
| 20:4n-6 ARA ^g^ | 0.58 | 0.98 | 1 | 1.19 |
| 22:4n-6 | 0.15 | 0 | 0 | 0 |
| 22:5n-6 | 0.25 | 5.3 | 5.44 | 6.29 |
| Total n-6 PUFA ^h^ | 24.75 | 29.09 | 30.23 | 30.46 |
| 18:3n-3 ALA ^i^ | 2 | 1.67 | 1.9 | 1.65 |
| 18:4n-3 | 0.62 | 0.13 | 0.24 | 0.19 |
| 20:3n-3 | 0.11 | 0 | 0 | 0.14 |
| 20:4n-3 | 0.41 | 0.35 | 0.33 | 0.38 |
| 20:5n-3 EPA ^j^ | 4.21 | 0.97 | 1.33 | 1.84 |
| 22:5n-3 | 0.99 | 0.19 | 0.21 | 0.24 |
| 22:6n-3 DHA ^k^ | 2.84 | 13.49 | 14.08 | 16.75 |
| Total n-3 PUFA ^l^ | 11.18 | 16.8 | 18.1 | 21.18 |
| Total PUFA | 35.93 | 45.89 | 48.33 | 51.65 |
| Total n-6 LCPUFA ^m^ | 8.76 | 15 | 15.96 | 19.34 |
| Total n-3 LCPUFA ^n^ | 1.42 | 6.69 | 6.83 | 7.76 |
| n-3/n-6 PUFA ratio ^o^ | 0.46 | 0.58 | 0.6 | 0.7 |
| n-3/n-6 LCPUFA ratio ^p^ | 6.18 | 2.24 | 2.34 | 2.49 |
| 20:5n-3 EPA/20:4n-6 ARA | 0.137 | 1.014 | 0.75 | 0.647 |

^a^  Reference: no replacement of fish meal (FM) and fish oil (FO).

^b^ Replacement of 33% of FM with *N. oculata* and 100% of FO with *Schizochytrium* sp.

^c^ Replacement of 66% of FM with *N. oculata* and 100% of FO with *Schizochytrium* sp.

^d^ Replacement of 100% of FM with *N. oculata* and 100% of FO with *Schizochytrium* sp.

^e^ Saturated fatty acids (SFA) is the sum of all fatty acids without double bonds.

^f^ Monounsaturated fatty acids (MUFA) is the sum of all fatty acids with a single bond.

^g^  Arachidonic acid (ARA).

^h^  Omega-6 (n-6) Polyunsaturated fatty acids (PUFAs) (sum of all fatty acids with ≥2 double bonds (18:2, 18:3, 20:2, 20:3, 20:4,

22:4, 22:5).

^i^ Alpha-linolenic acid (ALA).

^j^ Eicosapentaenoic acid (EPA).

^k^ Docosahexaenoic acid (DHA).

^l^ Omega-3 (n-3) PUFAs (18:3, 18:4, 20:3, 20:4, 20:5, 22:5, 22:6).

^m^ n-6 long-chain (LC) PUFA (20:2, 20:3, 20:4, 22:4, 22:5).

^n^ n-3 LCPUFA(20:3, 20:4, 20:5, 22:5, 22:6).

^o^ Ratio calculated for total n-3 PUFA: total n-6 PUFA (n-3/n-6).

^p^ Ratio calculated for total n-3 LCPUFA: total n-6 LCPUFA (n-3/n-6).

**Table S12.** Data sources, price regions and periodicity of commodities.

| Commodity | | Price region | | Periodicity | | | Source | |  |
| --- | --- | --- | --- | --- | --- | --- | --- | --- | --- |
| Soybean meal | | USA (IA, IL) | | Monthly | | | ^14^ | |  |
| Cottonseed meal | | USA (CA, MO, TN) | | Monthly | | | ^14^ | |  |
| Linseed meal | | USA (MN) | | Monthly ^a^ | | | ^14^ | |  |
| Canola (Rapeseed) meal | | International (Germany, Port of Hamburg) | | Monthly | | | ^13^ | |  |
| Corn gluten feed meal | | USA (NE, IA, IL, IN, MO) | | Monthly | | | ^14^ | |  |
| Fish meal | | International (Germany, Port of Bremen) | | Monthly | | | ^13^ | |  |
| Palmkernel meal | | International (Netherlands, Port of Rotterdam) | | Monthly | | | ^13^ | |  |
| Sunflower meal | USA (ND) | | Monthly | |  | ^14^ | |  |  |
| Oats | | USA (OR) | | Monthly ^b^ | | | ^14^ | |  |
| Barley | | International (Australia, Eastern States) | | Monthly | | | ^13^ | |  |
| Wheat flour | | USA (MN) | | Quarterly ^c^ | | | ^15^ | |  |
| Wheat middlings | | USA (IA, IL, MO, TN) | | Monthly | | | ^14^ | |  |
| Sunflower oil | | International (Northwest European Ports) | | Monthly | | | ^13^ | |  |
| Soybean oil | | USA (IA, IL, MN) | | Monthly | | | ^14^ | |  |
| Corn oil crude | | USA (IL) | | Monthly | | | ^14^ | |  |
| Fish oil | | International (Northwest European Ports) | | Monthly | | | ^13^ | |  |
| Canola (rapeseed) oil | | International (Netherlands, Port of Rotterdam) | | Monthly | | | ^13^ | |  |
| Coconut oil | | International (Phillipines, Port of Rotterdam) | | Monthly | | | ^13^ | |  |
| Palm oil | | International (Netherlands, Port of Rotterdam) | | Monthly | | | ^13^ | |  |

^a^  We applied the Producer Price Index (PPI) for Grains (<https://fred.stlouisfed.org/series/WPU012>) to the September 2012 price

to obtain prices between October, 2012 and December, 2019.

^b^ We applied the PPI for Grains to the October, 2012 price to obtain prices between January, 2010 and September, 2012; we

applied the PPI for Grains to the May, 2019 price to obtain prices between June, 2019 to December, 2019.

^c^ Quarterly data was available. To obtain monthly data we applied the PPI for Grains to quarterly data. We assumed the first

quarter price was the mean price for the month of February, the second quarter price was for the month of April, the third

quarter price was for the month of July, and the fourth quarter price was for the month of October.

**Table S13.** Origin and destination cities and distances by modal mix for commodities produced in the U.S.

| Commodity | Origin ^a^ | Destination ^b^ | Total distance (km) ^c^ | Rail (km) | Barge (km) | Truck (km) |
| --- | --- | --- | --- | --- | --- | --- |
| Soybean meal ^d^ | Central Illinois, IL | Port of New Orleans, LA | 1318 | 409 | 659 | 250 |
| Soybean meal | Iowa City, IA | Port of New Orleans, LA | 1483 | 460 | 742 | 282 |
| Soybean meal | Chicago, IL | Port of New Orleans, LA | 1489 | 462 | 744 | 283 |
| Soybean meal | Ohio, IL | Port of New Orleans, LA | 1469 | 455 | 734 | 279 |
| Cottonseed meal ^e^ | Fresno, CA ^f,g^ | Port of Los Angeles, CA | 355 | 131 | 167 | 57 |
| Cottonseed meal | St. Louis, MO | Port of New Orleans, LA | 1088 | 403 | 512 | 174 |
| Cottonseed meal | Memphis, TN | Port of New Orleans, LA | 636 | 235 | 299 | 102 |
| Linseed meal ^h^ | Duluth, MN | Port of New Orleans, LA | 2182 | 1200 | 720 | 262 |
| Corn gluten feed meal ^l^ | Des Moines, IA ^i, j, k,l^ | Port of New Orleans, LA | 1627 | 472 | 944 | 212 |
| Corn gluten feed meal | St. Louis, MO | Port of New Orleans, LA | 1088 | 316 | 631 | 141 |
| Sunflower meal ^e^ | Linton, ND ^m,n,o^ | Port of Tacoma, WA | 2107 | 779 | 990 | 337 |
| Oats ^e^ | Portland, OR | Port of Tacoma, WA | 232 | 86 | 109 | 37 |
| Wheat flour ^h^ | Kansas City, MO | Port of New Orleans, LA | 1358 | 747 | 448 | 163 |
| Wheat flour | Minneapolis, MN | Port of New Orleans, LA | 2182 | 1200 | 720 | 262 |
| Wheat middlings ^h^ | Chicago, IL | Port of New Orleans, LA | 1489 | 819 | 491 | 179 |
| Wheat middlings | Kansas City, MO | Port of New Orleans, LA | 1358 | 747 | 448 | 163 |
| Wheat middlings | St. Louis, MO | Port of New Orleans, LA | 1088 | 599 | 359 | 131 |
| Wheat middlings | Duluth, MN | Port of New Orleans, LA | 2182 | 1200 | 720 | 262 |
| Soybean oil ^d^ | Ohio, IL | Port of New Orleans, LA | 1469 | 455 | 734 | 279 |
| Soybean oil | Minnesota, MN | Port of New Orleans, LA | 2219 | 688 | 1110 | 422 |
| Soybean oil | Central Illinois, IL | Port of New Orleans, LA | 1318 | 409 | 659 | 250 |
| Soybean oil | Iowa City, IA | Port of New Orleans, LA | 1483 | 460 | 742 | 282 |
| Corn oil crude ^i^ | Central Illinois, IL | Port of New Orleans, LA | 1318 | 382 | 764 | 171 |

^a^ Source: ^14^

^b^ Source: ^1^

^c^ Distances estimated with Google maps.

^d^ Modal mix for soy products: 31% rail; 50% barge; 19% truck ^17^.

^e^ Modal mix for average grain product: 37% rail; 47% barge; 16% truck^17^.

^f^ Origin location listed as ‘Central San Joaquin Valley, CA’.

^g^ We selected Fresno as origin city because it is a top producer of cotton^18^.

^h^ Modal mix for wheat products: 55% rail; 33% barge; 12% truck^17^.

^i^ Modal mix for corn products: 29% rail; 58% barge; 13% truck^17^.

^j^ Origin location listed as ‘Midwest (NE, IA, IL, IN), MO’.

^k^ The leading corn producing state is Iowa^19^.

^l^ We selected Des Moines as origin city because it is a top producer of corn^20^.

^m^ Origin location listed as ‘North Dakota/Minnesota, CO’.

^n^ North Dakota is a top exporter of sunflower products^21^.

^o^ Emmons County is the leading producer of sunflower for oil^21^. We selected Linton as origin city because it is the largest city

in Emmons County.

**Table S14.** International shipping distances of commodities used to estimate the ocean freight costs.

| Commodity | Origin | Destination | Total distance (km) ^a^ |
| --- | --- | --- | --- |
| Soybean meal | Port of New Orleans | Port of Shanghai | 18544 |
| Cottonseed meal | Port of Los Angeles | Port of Shanghai | 10571 |
| Linseed meal | Port of New Orleans | Port of Shanghai | 18544 |
| Canola (Rapeseed) meal | Port of Hamburg | Port of Shanghai | 19961 |
| Corn gluten feed meal | Port of New Orleans | Port of Shanghai | 18544 |
| Fish meal ^b^ | Port of Bremen | Port of Shanghai |  |
| Palmkernel meal ^c^ | Port of Rotterdam | Port of Shanghai |  |
| Sunflower meal | Port of Tacoma | Port of Shanghai | 9471 |
| Oats | Port of Tacoma | Port of Shanghai | 9471 |
| Barley | Port of Brisbane | Port of Shanghai | 7836 |
| Wheat flour | Port of New Orleans | Port of Shanghai | 18544 |
| Wheat middlings | Port of New Orleans | Port of Shanghai | 18544 |
| Sunflower oil | Port of Rotterdam | Port of Shanghai | 19492 |
| Soybean oil | Port of New Orleans | Port of Shanghai | 18544 |
| Corn oil crude | Port of New Orleans | Port of Shanghai | 18544 |
| Fish oil ^c^ | North West Europe | Port of Shanghai |  |
| Canola (rapeseed) oil | Port of Rotterdam | Port of Shanghai | 19492 |
| Coconut oil ^c^ | Port of Rotterdam | Port of Shanghai |  |
| Palm oil ^c^ | Port of Rotterdam | Port of Shanghai |  |

^a^ Shipping distances estimated with a sea route calculator (available at <https://sea-distances.org/>).

^b^ Price is given 'f.c.a'; according to the f.c.a. Incoterm definition, it is the seller’s responsibility to deliver the goods

https://www.icontainers.com/help/incoterms/fca/). Thus, additional shipping charges were not added.

^c^ Price is given for 'c.i.f.'; according to the c.i.f. Incoterm definition, the seller delivers the goods, cleared for export, onboard the

vessel at the port of shipment, pays for the transport of the goods to the port of destination, and also obtains and pays for

minimum insurance coverage on the goods through their journey to the named port of destination. Thus, additional shipping

charges were not added.

**Table S15.** Key nutritional components of commodities used as aquafeed meals.

| Ingredient ^a^ | Crude protein | Ether extract | Methionine | Lysine | Dry matter | Source |
| --- | --- | --- | --- | --- | --- | --- |
| Corn gluten meal | 0.602 | 0.02 | 0.0143 | 0.0102 | 0.9 | ^22^ |
| Soybean meal | 0.475 | 0.01 | 0.0068 | 0.0309 | 0.88 | ^22^ |
| Cotton seed meal | 0.414 | 0.0105 | 0.0067 | 0.0172 | 0.9 | ^22^ |
| Canola meal | 0.365 | 0.035 | 0.0073 | 0.0216 | 0.93 | ^22^ |
| Sunflower meal (30%) | 0.3 | 0.0103 | 0.0069 | 0.0112 | 0.9 | ^22^ |
| Linseed meal | 0.36 | 0.02 | 0.0059 | 0.0124 | 0.9 | ^22^ |
| Wheat middlings | 0.145 | 0.042 | 0.0025 | 0.0064 | 0.89 | ^22^ |
| Barley | 0.105 | 0.019 | 0.0017 | 0.0036 | 0.89 | ^22^ |
| Fish meal | 0.652 | 0.092 | 0.0177 | 0.0489 | 0.921 | ^23^ |
| Wheat flour | 0.134 | 0.024 | 0.002 | 0.005 | 0.877 | ^24^ |
| Palm kernel meal | 0.156 | 0.075 | 0.0028 | 0.0046 | 0.916 | ^25^ |
| Oats | 0.094 | 0.047 | 0.0017 | 0.004 | 0.876 | ^26^ |

^a^ As-is basis.

**Table S16.** Fatty acid characteristics of commodity oils used in the hedonic analysis.

| Commodity | 14:0 | 16:0 | 16:1n-7 | 20:5n-3 |
| --- | --- | --- | --- | --- |
| Canola oil | 0.001 | 0.051 | 0.002 | 0 |
| Coconut oil | 0.185 | 0.087 | 0 | 0 |
| Corn oil | 0 | 0.123 | 0.001 | 0 |
| Fish oil | 0.081 | 0.179 | 0.139 | 0.149 |
| Palm oil | 0.011 | 0.438 | 0.002 | 0 |
| Soybean oil | 0.001 | 0.108 | 0.002 | 0 |
| Sunflower oil | 0.001 | 0.064 | 0.001 | 0 |

**Table S17.** Scale parameters applied to vectors of independent variables in defatted N. oculata meal model.

| Scale parameter | **CP** | **EE** | **Met** | **Lys** |
| --- | --- | --- | --- | --- |
| Mean | 0.3175 | 0.033058 | 0.006375 | 0.014692 |
| Standard Deviation | 0.185611 | 0.025794 | 0.004847 | 0.012976 |

**Table S18.** Scale parameters applied to vectors of independent variables in Schizochytrium sp. model.

| Scale parameter | **14:0** | **16:0** | **16:1n-7** | **20:5n-3** |
| --- | --- | --- | --- | --- |
| Mean | 0.04 | 0.15 | 0.021 | 0.021286 |
| Standard Deviation | 0.065148 | 0.123892 | 0.048178 | 0.052139 |

**Table S19.** Results of the Levene test of the defatted N. oculata meal model.

| Fixed coefficient (response variable) | Degrees of Freedom | Sum Sq | Mean Sq | F value | P value |
| --- | --- | --- | --- | --- | --- |
| β_1_ (**CP**^2^) | 1 | 6639600 | 6639600 | 0.1034 | 0.7484 |
| β_2_ (**Met**^2^) | 1 | 79294645 | 79294645 | 1.2347 | 0.2688 |
| β_2_ (**Lys**^2^) | 1 | 2727396 | 2727396 | 0.0425 | 0.8371 |
| β_3_ (**EE)** | 1 | 225128523 | 225128523 | 3.5056 | 0.0637 |

**Table S20.** Results of the Levene test for the Schizochytrium sp. oil

| Response variable | Degrees of Freedom | Sum Sq | Mean Sq | f-value | p-value |
| --- | --- | --- | --- | --- | --- |
| β_1_ (**20:5n-3**^2^) | 1 | 23423577 | 23423577 | 3.7279 | 0.05794 |
| β_2_ (**14:0**^2^) | 1 | 30083623 | 30083623 | 4.7879 | 0.03232 |
| β_3_ (**16:1n-7**^2^) | 1 | 8287323 | 8287323 | 1.3189 | 0.25506 |
| β_4_ (**14:0**) | 1 | 25414328 | 25414328 | 4.0447 | 0.04853 |
| β_5_ (**16:0**) | 1 | 192675 | 192675 | 0.0307 | 0.86154 |

**Table S21.** Scaled independent variables used in hedonic regression analysis of defatted N. oculata meal.

| Commodity | **CP** | **EE** | **Met** | **Lys** |
| --- | --- | --- | --- | --- |
| Barley | -1.14487 | -0.85905 | -0.96444 | -0.8548 |
| Canola meal | 0.255912 | 0.075276 | 0.190826 | 0.532402 |
| Cottonseed meal | 0.519905 | -0.87456 | 0.067047 | 0.193309 |
| Corn gluten feed meal | 1.532776 | -0.50626 | 1.634911 | -0.34616 |
| Fishmeal | 1.802157 | 2.2851 | 2.336324 | 2.636323 |
| Lindseed meal | 0.228974 | -0.50626 | -0.09799 | -0.17661 |
| Oats | -1.20413 | 0.540502 | -0.96444 | -0.82397 |
| Palm kernel meal | -0.827 | 1.62603 | -0.69626 | -0.75461 |
| Soybean meal | 0.848549 | -0.89394 | 0.087677 | 1.249122 |
| Sunflower meal | -0.09428 | -0.88231 | 0.108306 | -0.26909 |
| Wheat flour | -0.98863 | -0.35118 | -0.90255 | -0.7469 |
| Wheat middlings | -0.92936 | 0.346658 | -0.7994 | -0.63901 |
| Defatted N. oculata meal ^a^ | 0.967077 | 0.579271 | 0.74783 | 0.948562 |

^a^ Values were not included in the hedonic regression.**Table S22.** Fixed-effect coefficients of the defatted *N. oculata* meal model.

| Fixed effect coefficient ^a^ | Estimate | Standard Error | Degrees of Freedom | t-value | p-value |
| --- | --- | --- | --- | --- | --- |
| β_0_ (Intercept) | 198.144 | 16.739 | 24.93 | 11.837 | 9.94E-12 |
| β_1_ (**CP**^2^) | -96.997 | 23.618 | 105.597 | -4.107 | 7.93E-05 |
| β_2_ (**Met**^2^) | 159.38 | 19.441 | 107.535 | 8.198 | 5.61E-13 |
| β_3_ (**Lys**^2^) | 116.669 | 7.612 | 98.887 | 15.328 | < 2e-16 |
| β_4_ (**EE**) | -717.64 | 10.259 | 103.813 | -6.98 | 2.87E-10 |

^a^ With the exception of the intercept, the associated vector of independent variables given in parenthesis.

**Table S23.** Random-effect coefficients of the defatted N. oculata meal model.

| t(Year) | b_0,CPt_ (Intercept) | b_1t_ (**CP**) ^a^ | b_0,EEt_ (Intercept) | b_2t_  (**EE**) ^a^ | Residual |
| --- | --- | --- | --- | --- | --- |
| 2010 | 80.1865 | 60.19609 | -11.74181 | 2.0462254 | 68.383 |
| 2011 | 59.08408 | 44.35448 | 8.0452096 | -1.402025 | 68.383 |
| 2012 | 95.328 | 71.56283 | 13.431986 | -2.340769 | 68.383 |
| 2013 | 123.09856 | 92.41021 | 6.0818841 | -1.059879 | 68.383 |
| 2014 | 124.30109 | 93.31295 | 2.4369459 | -0.424682 | 68.383 |
| 2015 | 66.606 | 50.00119 | -5.054175 | 0.8807823 | 68.383 |
| 2016 | 42.78643 | 32.11982 | -11.98108 | 2.0879222 | 68.383 |
| 2017 | 24.81587 | 18.6293 | -3.403062 | 0.5930456 | 68.383 |
| 2018 | 56.27681 | 42.24706 | 0.9362837 | -0.163165 | 68.383 |
| 2019 | 29.23704 | 21.94827 | 1.2478233 | -0.217456 | 68.383 |

^a^ The associated vector of independent variables given in parenthesis.

**Table S24.** Scaled independent variables used in hedonic regression analysis of Schizochytrium sp. oil.

| Commodity | **14:0** | **16:0** | **16:1n-7** | **20:5n-3** |
| --- | --- | --- | --- | --- |
| Canola oil | -0.59864 | -0.79909 | -0.39437 | -0.40825 |
| Coconut oil | 2.225695 | -0.50851 | -0.43588 | -0.40825 |
| Corn oil | -0.61398 | -0.21793 | -0.41513 | -0.40825 |
| Fish oil | 0.629335 | 0.234075 | 2.449238 | 2.44949 |
| Palm oil | -0.44514 | 2.324612 | -0.39437 | -0.40825 |
| Soybean oil | -0.59864 | -0.33901 | -0.39437 | -0.40825 |
| Sunflower oil | -0.59864 | -0.69415 | -0.41513 | -0.40825 |
| *Schizochytrium* sp. oil ^a^ | 0.81353 | 0.758727 | -0.39437 | -0.25481 |

^a^ Values were not included in the hedonic regression.

**Table S25.** Fixed-effect coefficients of the Schizochytrium sp. oil model.

| Fixed effect coefficient ^a^ | Estimate | Standard Error | Degrees of Freedom | t-value | p-value |
| --- | --- | --- | --- | --- | --- |
| β_0_ (Intercept) | 3095.96 | 289.43 | 43.86 | 10.697 | 8.37E-14 |
| β_1_ (**20:5n-3**^2^) | -3471.54 | 774.14 | 40.31 | -4.484 | 5.95E-05 |
| β_2_ (**14:0**^2^) | -1613.34 | 213.04 | 39.71 | -7.573 | 3.17E-09 |
| β_3_ (**16:1n-7**^2^) | 3069.35 | 741.69 | 40.04 | 4.138 | 0.000175 |
| β_4_ (**14:0**) | 2548.15 | 345.81 | 40.68 | 7.822 | 1.23E-09 |
| β_5_ (**16:0**) | -278.62 | 28.99 | 39.97 | -9.611 | 6.03E-12 |

^a^ With the exception of the intercept, the associated vector of independent variables given in parenthesis.

**Table S26.** Random-effect coefficients of the Schizochytrium sp. oil model.

| t (Year) | b_0,14:0t_ (Intercept) | b_1t_ (**14:0**) ^a^ | b_0,16:0t_ (Intercept) | b_2t_ (**16:0**) ^a^ | b_0,16:1n-7t_ (Intercept) | b_3t_ (**16:1n-7**) ^a^ | Residuals |
| --- | --- | --- | --- | --- | --- | --- | --- |
| 2010 | -144.415 | 147.5599 | -15.8679 | -1.0298 | 197.4762 | -272.653 | 52.764 |
| 2011 | 117.4442 | -120.002 | 22.6206 | 1.468053 | 88.89916 | -122.72 | 52.764 |
| 2012 | 213.1389 | -217.78 | -1.57803 | -0.10243 | -53.227 | 73.49018 | 52.764 |
| 2013 | 87.13678 | -89.0343 | 94.87672 | 6.157411 | -85.4165 | 117.9914 | 52.764 |
| 2014 | -10.4165 | 10.64337 | 82.11203 | 5.329061 | -62.8973 | 86.87216 | 52.764 |
| 2015 | -2.3719 | 2.423534 | -37.0036 | -2.40151 | -66.8949 | 92.33627 | 52.764 |
| 2016 | -83.7483 | 85.57209 | 27.85276 | 1.807645 | -6.34277 | 8.767485 | 52.764 |
| 2017 | -157.918 | 161.3571 | 23.3038 | 1.512415 | 77.07775 | -106.406 | 52.764 |
| 2018 | -89.7776 | 91.73263 | -24.1275 | -1.56588 | -7.90917 | 10.91095 | 52.764 |
| 2019 | 70.92765 | -72.4723 | -172.189 | -11.175 | -80.7652 | 111.4108 | 52.764 |

^a^ The associated vector of independent variables given in parenthesis.

**Table S27.** Median [and 95% confidence interval] price of formulated feed by ingredient.

| Ingredient | Reference ^a^ | 33NS ^b^ | 66NS ^c^ | 100NS ^d^ |
| --- | --- | --- | --- | --- |
|  | ($ / kg ingredient) | | | |
| Fish meal | 0.108 [0.101, 0.114] | 0.072 [0.068, 0.076] | 0.037 [0.034, 0.039] | 0 |
| *N. oculata* co-product | 0 | 0.013 [0.012, 0.015] | 0.024 [0.022, 0.027] | 0.035 [0.032, 0.039] |
| Fish oil | 0.054 [0.052, 0.056] | 0 | 0 | 0 |
| *Schyzochytrium* sp. | 0 | 0.148 [0.120, 0.159] | 0.148 [0.120, 0.159] | 0.148 [0.120, 0.159] |
| Corn gluten meal | 0.185 [0.177, 0.198] | 0.185 [0.177, 0.198] | 0.185 [0.177, 0.198] | 0.185 [0.177, 0.198] |
| Soybean meal | 0.141 [0.137, 0.151] | 0.141 [0.137, 0.151] | 0.141 [0.137, 0.151] | 0.141 [0.137, 0.151] |
| Wheat flour | 0.093 [0.088, 0.102] | 0.093 [0.088, 0.102] | 0.093 [0.088, 0.102] | 0.093 [0.088, 0.102] |
| Dicalcium Phosphate (21%) | 0.003 [0.002, 0.003] | 0.003 [0.002, 0.003] | 0.003 [0.002, 0.003] | 0.003 [0.002, 0.003] |
| L-lysine HCl | 0 | 0.008 [0.007, 0.009] | 0.009 [0.007, 0.010] | 0.010 [0.008, 0.011] |
| DL-Methionine | 0 | 0.005 [0.003, 0.008] | 0.005 [0.003, 0.009] | 0.005 [0.003, 0.009] |
| Choline chloride | 0.016 [0.008, 0.018] | 0.016 [0.008, 0.018] | 0.016 [0.008, 0.018] | 0.016 [0.008, 0.018] |
| Mineral mix | 0.006 | 0.006 | 0.006 | 0.006 |
| Vitamin mix | 0.035 | 0.035 | 0.035 | 0.035 |

^a^  Reference: no replacement of fish meal (FM) and fish oil (FO).

^b^ Replacement of 33% of FM with *N. oculata* and 100% of FO with *Schizochytrium* sp.

^c^ Replacement of 66% of FM with *N. oculata* and 100% of FO with *Schizochytrium* sp.

^d^ Replacement of 100% of FM with *N. oculata* and 100% of FO with *Schizochytrium* sp.

**Fig. S1.** Growth curves for each treatment from day 0 to day 184 for four different diets: No replacement of fish meal (FM) and fish oil (FO) (0NS); replacement of 33% of FM with *N. oculata* and 100% of FO with *Schizochytrium* sp. (33NS); replacement of 66% of FM with *N. oculata* and 100% of FO with *Schizochytrium* sp. (66NS); and replacement of 100% of FM with *N. oculata* and 100% of FO with *Schizochytrium* sp. (100NS). Linear regression equations and coefficient of determination (R^2^) values: 0NS, y = 0.5563x + 46.321(R² = 0.9229); 33NS, y = 0.842x + 44.173 (R² = 0.9763); 66NS, y = 0.7036x + 42.747 (R² = 0.9716); and 100NS, y = 0.9176x + 41.13 (R² = 0.978). Error bars show standard error of the mean.


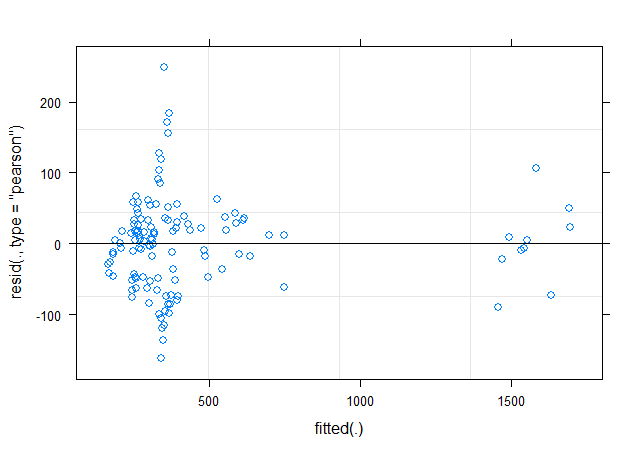


**Fig. S2.** Plot of residuals (the difference between the observed value and the model-estimated value) vs the predictor of the defatted N. oculata meal model.


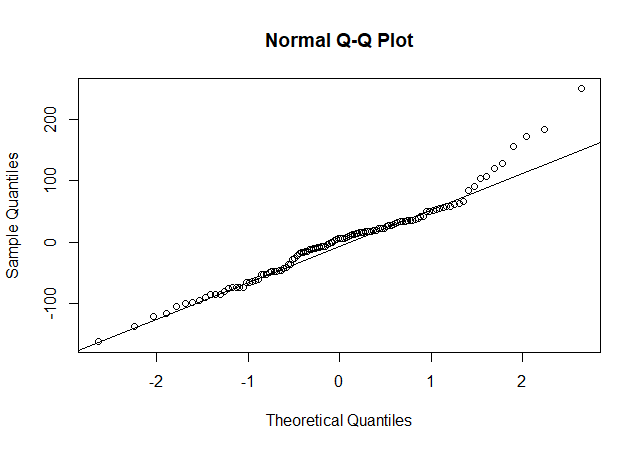


**Fig. S3.** Plot of the standardized residuals versus the standard normal quantiles of the defatted N. oculata meal model.

**Fig. S4.** Actual vs. predicted values of the defatted N. oculata meal model.

**
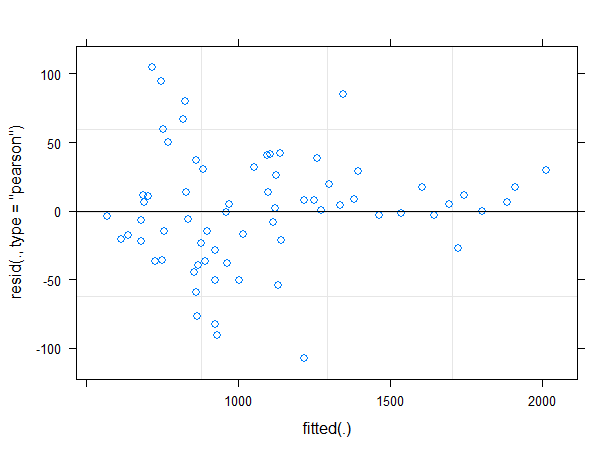
Fig. S5.** Plot of residuals (the difference between the observed value and the model-estimated value) vs the predictor of the Schizochytrium sp. oil model.


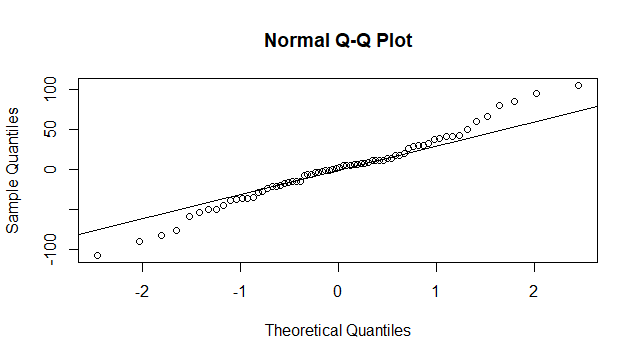


**Fig. S6.** Plot of the standardized residuals versus the standard normal quantiles of the Schizochytrium sp. oil model.

**Fig. S7.** Actual vs. predicted values of the Schizochytrium sp. oil model.

**References**

1. USDA, Profiles of Top U.S. Agricultural Ports, Agricultural Marketing Service, United States

Department of Agriculture, (2017). Available at:

<https://www.ams.usda.gov/sites/default/files/media/PortProfilesMap2019.pdf>.

2. Bureau of Transportation Statistics, Table 3-21: Average freight revenue per ton-mile. United States

of Department of Transportation, Bureau of Transportation (2019). Available at:

<https://www.bts.gov/archive/publications/national_transportation_statistics/table_03_21>.

3. USDA, Bulk ocean freight rates, Agricultural Marketing Service, United States Department of

Agriculture (2020). Available at:

[https://agtransport.usda.gov/Bulk/Bulk-Grain-Ocean-Freight- Rates/ehic-wtxb](https://agtransport.usda.gov/Bulk/Bulk-Grain-Ocean-Freight-%20%20%20%20%20Rates/ehic-wtxb).

4. Nakagawa, S. & Schielzeth, H. A general and simple method for obtaining *R*^2^ from generalized linear

mixed‐effects models. *Methods Ecol Evol*, **4**: 133-142 (2013).

5. Barto, K. Multi-Model Inference package in R (MuMIn v1.40.0) (2017). Aavailable at:

<https://www.rdocumentation.org/packages/MuMIn>.

6. Fox, J. *Applied Regression Analysis and Generalized Linear Models, 3rd Edition*. (Sage: Thousand

Oaks, CA, 2016).

7. Palmeri, M. Testing the assumptions of multi-level models. In, *A Language Not A Letter: Learning*

*Statistics in R* (University of Illinois at Chicago, 2016). Available at:

<https://ademos.people.uic.edu/Chapter18.html>.

8. Dunn, P. & Smyth, G. *Generalized Linear Model with Examples in R* (Springer Science+Business

Media, LLC, part of Springer Nature, New York, N.Y., U.S.A, 2018).

9. Bryant, H.L. *et al.* The value of post-extracted algae residue. *Algal Res* 1: 185–193. (2012).

10. FAO, FAO Yearbook. Fishery and Aquaculture Statistics 2017. (Food and Agriculture Organization

(FAO) of the United Nations, 2019).

11. Jackson, A. Fish In-Fish Out ratios explained. *Aquaculture Europe* **34** **(3)**: 34: 5-10 (2009).

12. Tacon, A. Trends in global aquaculture and aquafeed production: 2000–2017, *Reviews in Fisheries*

*Science & Aquaculture* (2019).

13. FAO, GIEWS FMPA Tool (v.3.8.0): monitoring and analysis of food prices (Food and Agriculture

Organization (FAO) of the United Nations, 2020). Available at:

[https://fpma.apps.fao.org/giews/food- prices/tool/public/#/dataset/international](https://fpma.apps.fao.org/giews/food-%20prices/tool/public/#/dataset/international).

14. USDA, Agricultural marketing service, Custom reports (United States Department of Agriculture,

2020). Available at: <https://marketnews.usda.gov/mnp/ls-report-config>.

15. USDA, Wheat Data, Economic Research Service, (United States Department of Agriculture, 2020)

Available at: <https://www.ers.usda.gov/data-products/wheat-data/>.

16. Alibaba, Product searches, Web accessed Nov. 4, 2019. Available at: <https://www.alibaba.com/>.

17. Chang, K.-L., *et al.* Transportation of U.S. Grains: A Modal Share

Analysis. Agricultural Marketing Service, (United States Department of Agriculture, 2019).

18. California Department of Food and Agriculture California Agricultural Statistics Review 2017-2018

(2019). Available at: <https://www.cdfa.ca.gov/statistics/PDFs/2017-18AgReport.pdf>.

19. USDA, Farm income and wealth statistics: Cash receipts by commodity, State ranking, 2018,

Economic Research Service (United States Department of Agriculture, 2020).

20. USDA, 2018 Iowa Agricultural Statistics. National Agricultural Statistics Service Upper Midwest Reg

ional Office, (United States Department of Agriculture, 2018).

21. USDA, 2018 North Dakota Agricultural Statistics. National Agricultural Statistics Service Upper Mid

west Regional Office, (United States Department of Agriculture, 2018).

22. Archer Daniels Midland, Ingredients Catalog Feed and Pet Food, (Archer Daniels Midland, 2016).

23. Feedinamics, Feed tables: Fish meal, protein 65% (2019). Available at:

<https://www.feedtables.com/content/fish-meal-protein-65>.

24. Feedinamics, Feed tables: Wheat feed flour (2019). Available at:

<https://feedtables.com/content/wheat-feed-flour>).

25. Feedinamics, Feed tables: Palm kernel meal, oil 5-20% (2019). Available at:

<https://feedtables.com/content/palm-kernel-meal-oil-5-20>.

26. Feedinamics, Feed tables: Oats (2019). Available at: <https://feedtables.com/content/oats>.
